# *Guttigomphus avilionis* gen. et sp. nov., a trirachodontid cynodont from the upper *Cynognathus* Assemblage Zone, Burgersdorp Formation of South Africa

**DOI:** 10.7717/peerj.14355

**Published:** 2022-12-16

**Authors:** Romy R. Rayner, Richard J. Butler, Christian F. Kammerer, Jonah N. Choiniere

**Affiliations:** 1 School of Geography, Earth & Environmental Sciences, University of Birmingham, Birmingham, United Kingdom; 2 Evolutionary Studies Institute, University of the Witwatersrand, Johannesburg, South Africa; 3 North Carolina Museum of Natural Sciences, Raleigh, North Carolina, United States of America

**Keywords:** Middle Triassic, South Africa, Phylogenetics, Taxonomy, Anatomy, Anisian, Cynodont, Synapsid, Burgersdorp Formation, Trirachodontid

## Abstract

The Burgersdorp Formation of South Africa is a richly fossiliferous rock sequence at the top of the Permian–Triassic Beaufort Group and is known for its abundance of Early–Middle Triassic vertebrate remains, particularly cynodonts. Fossils from the Burgersdorp Formation are referred biostratigraphically to the *Cynognathus* Assemblage Zone (CAZ), which is further divided into three subzones: *Langbergia*-*Garjainia, Trirachodon*-*Kannemeyeria*, and *Cricodon-Ufudocyclops*. Each subzone is characterised by the presence of a distinct species of trirachodontid, a group of gomphodont cynodonts found relatively abundantly throughout the CAZ, with the lower two subzones characterised by the medium-sized trirachodontids *Langbergia* and *Trirachodon*. The uppermost part of the formation, the *Cricodon-Ufudocyclops* subzone, yields trirachodontids of larger size. The majority of these trirachodontid specimens have previously been referred to *Cricodon metabolus*, a taxon also known from the Manda Beds of Tanzania and the Ntawere Formation of Zambia. Here we identify one of the specimens (BP/1/5538) previously referred to *Cricodon* as a new taxon, *Guttigomphus avilionis*. *Guttigomphus* can be distinguished from other gomphodont cynodonts by features of the upper postcanine teeth, such as an asymmetric crown in occlusal view (crown narrower along the lingual margin than the labial). Our phylogenetic analysis recovers *Guttigomphus* as a basal member of Trirachodontidae, outside of the clade including *Cricodon*, *Langbergia* and *Trirachodon*.

## Introduction

The Karoo Supergroup is a highly fossiliferous sequence of rocks that crops out across two thirds of the land area of present-day South Africa and preserves a time span stretching from the Carboniferous to the Early Jurassic (Schluter, 2008). In this span, it encompasses two mass extinctions, providing the best terrestrial record of the end-Permian mass extinction and the subsequent Triassic recovery period. The Permian–Triassic boundary occurs within the Beaufort Group, the most extensive subdivision of the Karoo Supergroup in terms of outcrop area and second most extensive in terms of geological time (since revision of the chronostratigraphy of the Stormberg Group; Bordy et al., 2020). Seven temporally distinct faunal assemblages are now recognized within the Beaufort Group (Smith et al., 2020), the youngest of which is the *Cynognathus* Assemblage Zone (CAZ). The CAZ has in turn been divided into three subunits. These were initially informally labeled subzones A–C and characterised largely by temnospondyl amphibian and cynodont index taxa (Hancox et al., 1995; Hancox, 1998, 2000). These subunits have now been formalized as the *Langbergia*-*Garjainia* (formerly subzone A), *Trirachodon*-*Kannemeyeria* (B), and *Cricodon*-*Ufudocyclops* (C) subzones (Hancox, Neveling & Rubidge, 2020), with trirachodontid cynodonts as the primary index taxa.

The Karoo Basin provides an excellent record of the early members of Cynodontia, the therapsid subclade that includes mammals. The earliest cynodonts currently known, *Abdalodon diastematicus* and *Charassognathus gracilis*, are known from rare remains in the Lopingian-aged (Wuchiapingian) Teekloof Formation (*Endothiodon* Assemblage Zone) (Botha, Abdala & Smith, 2007; Day et al., 2015; Kammerer, 2016; Day & Smith, 2020). Additional taxa, such as *Procynosuchus delaharpeae*, *Cynosaurus suppostus*, and the recently described *Vetusodon elikhulu*, are known from slightly younger (Wuchiapingian–Changhsingian) strata in the latest Permian *Cistecephalus–Daptocephalus* assemblage zones (Viglietti et al., 2016; Van den Brandt & Abdala, 2018; Abdala et al., 2019). In the earliest Triassic (Induan), additional cynodont taxa are found, including well-known species such as *Galesaurus planiceps* (Owen, 1859; Parrington, 1934; Pusch, Kammerer & Fröbisch, 2019) and *Thrinaxodon liorhinus* (Seeley, 1894; Estes, 1961; Jasinoski, Abdala & Fernandez, 2015).

**Figure 1:**
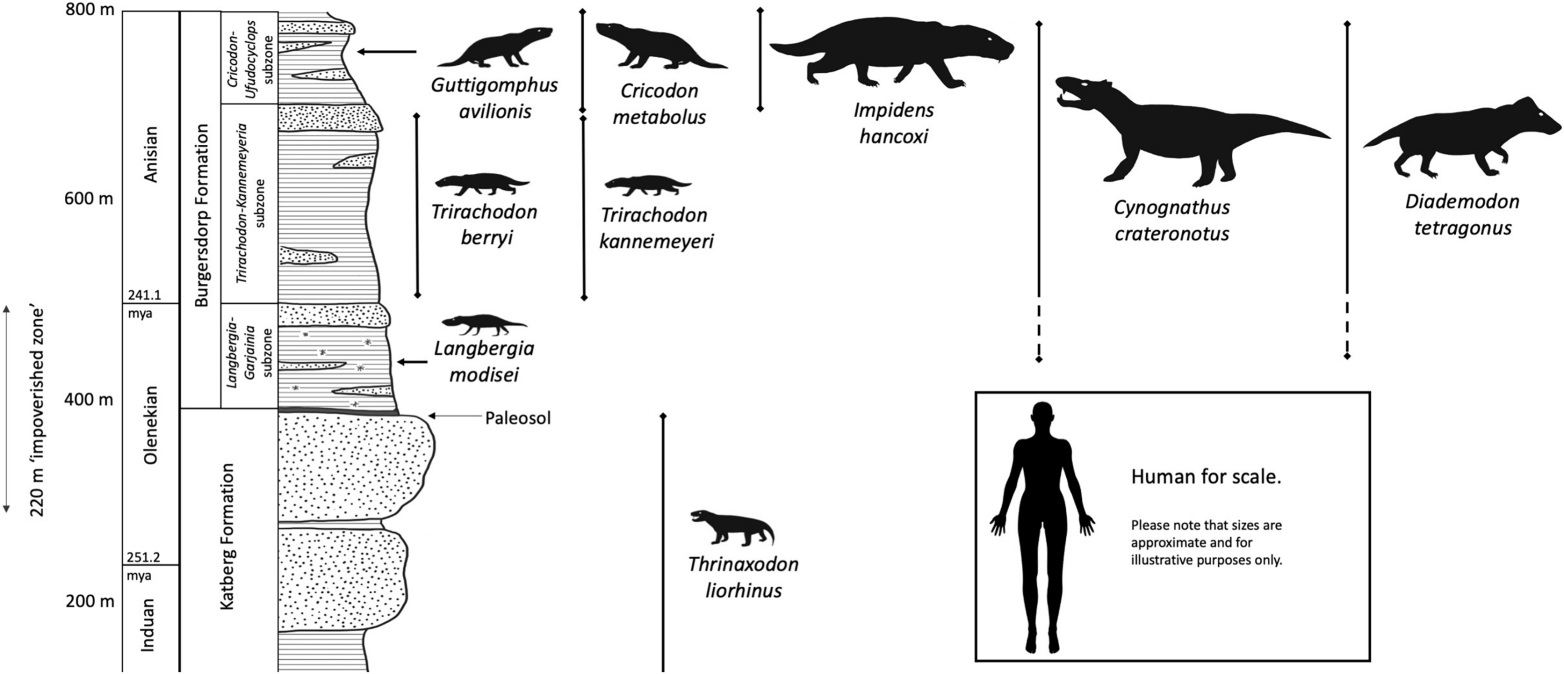
Lithology and ranges of key cynodont taxa from the Burgersdorp Formation and underlying Katberg Formation. This is a generalized interpretation based on the area around Sterkstroom and modified from the work of Hancox (1998), then updated based on information in Hancox, Neveling & Rubidge (2020). The palaeosol marking the boundary between the upper Katberg and lower Burgersdorp formations is only found across some of the outcrop. Taxa are shown approximately to scale in relation to each other. Silhouettes modified from life reconstructions by Mojca Janezic, Nobu Tamura, and Gabriel Ugueto.

The CAZ is currently thought to encompass the boundary between the late Lower (Olenekian) and early Middle (Anisian) Triassic (Hancox et al., 1995; although see Ottone et al. (2014) for an alternate proposal) and is characterised by abundant remains of the large-bodied cynognathian cynodonts *Cynognathus* and *Diademodon*. General concordance between the biostratigraphical CAZ (originally just “*Cynognathus* Zone”) and the lithostratigraphical Burgersdorp Formation has long been recognized (*e.g*., Watson, 1914), but the precise extent of the latter has been subject to some debate. Earlier works (*e.g*., Keyser & Smith, 1978; Groenewald, Kitching & Rubridge, 1995) considered the lowest parts of the Burgersdorp Formation to fall within the *Lystrosaurus* Assemblage Zone, with the CAZ corresponding to the rest of the formation. These studies recognised an ‘impoverished zone’ typified by fossil paucity at the boundary between the two assemblage zones (Fig. 1), corresponding to the “*Procolophon* Zone” of Broom (1906). This area of fossil paucity has also been proposed as an extinction event (Lucas, 1998), reflected in the abrupt faunal turnover existing between the two assemblages (Anderson & Cruickshank, 1978). However, more recent studies (Neveling, 2002, 2004) have revealed typical fossils for the CAZ in the ‘impoverished zone’ (*e.g*., the temnospondyl *Kestrosaurus* and trirachodontid cynodonts). Neveling, Hancox & Rubidge (2006) provided updated range data for tetrapod taxa in the ‘impoverished zone’, demonstrating that the entire Burgersdorp Formation correlates with the CAZ.

Hancox et al. (1995) initially proposed division of the CAZ into three subzones (A, B, and C), based primarily on the recognition of differences in the temnospondyl fauna. The lowest subzone (A) was characterised by the presence of the temnospondyl *Kestrosaurus* and also fossils of gomphodont cynodonts and an erythrosuchid archosauriform (later described as *Garjainia madiba* Gower et al., 2014). The middle subzone (B) is the most extensive (in thickness and area of exposure) and fossiliferous of the three. In addition to the temnospondyl index fossil *Xenotosuchus africanus* (see Morales & Shishkin, 2002), subzone B yields numerous cynodonts, bauriid therocephalians, the erythrosuchid *Erythrosuchus africanus*, and abundant remains of the kannemeyeriid dicynodont *Kannemeyeria*. Finally, the uppermost subzone (C) was originally considered to be characterised by “advanced capitosauroid amphibians” (Hancox et al., 1995; later described as *Paracyclotosaurus morganorum* Damiani & Hancox, 2003), archosaurs (although this has yet to be verified), and a “new tuskless kannemeyeriid dicynodont” (later referred to the Tanzanian taxon *Angonisaurus* (see Hancox & Rubidge, 1996), but more recently confirmed as a new taxon, *Ufudocyclops mukanelai* Kammerer et al., 2019).

Subsequently, additional support for the threefold subdivision of the CAZ has come from taxonomic revisions of the trirachodontid cynodont fauna (previously considered to represent a single genus, *Trirachodon*, throughout the CAZ; Hancox et al., 1995; Neveling, Hancox & Rubidge, 2006). Abdala, Hancox & Neveling (2005), Abdala, Neveling & Welman (2006) considered *Trirachodon* proper to be restricted to subzone B, recognising subzone A specimens as a new taxon (*Langbergia modisei*) and referring subzone C specimens to *Cricodon metabolus*, a species originally known from the Manda Beds of Tanzania (Crompton, 1955). Referral of the subzone C specimens to *Cricodon* was based primarily on their greater size compared to other CAZ trirachodontids and similarities in postcanine morphology (Abdala, Hancox & Neveling, 2005). This taxonomic scheme for trirachodontids underlies the recent formal nomenclature for the CAZ subunits (see above), which are based on co-occurrence of a particular trirachodontid genus with archosauromorph or dicynodont secondary index taxa (Hancox, Neveling & Rubidge, 2020). However, there is substantial variation in morphology within the subzone C specimens previously referred to *Cricodon metabolus*, which suggests that more than one taxon may be present (Hendrickx, Abdala & Choiniere, 2019).

**Figure 2:**
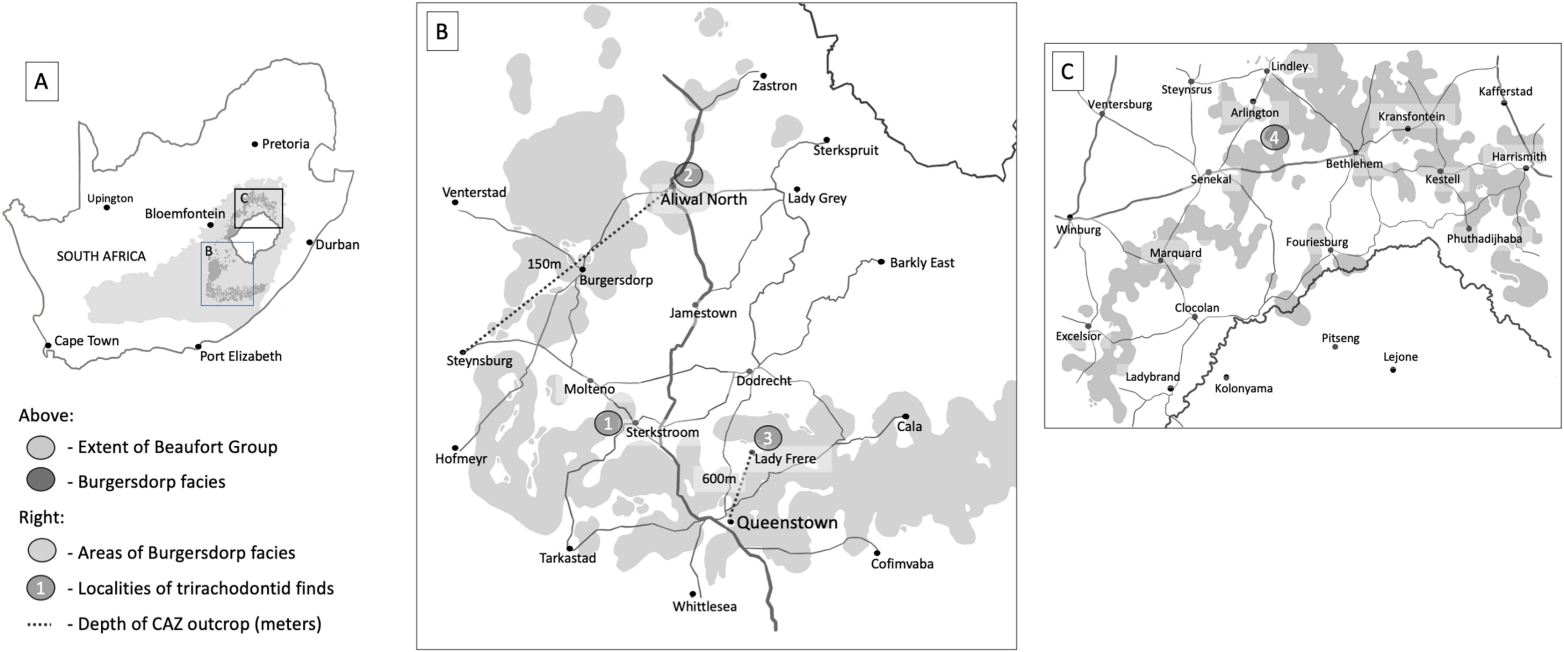
Areas of significant outcrops of the Burgersdorp Formation. (A) Map of South Africa showing the extent of the Burgersdorp facies and wider Beaufort Group along with the positioning of (B), the southern Burgersdorp facies and (C), the northern Burgersdorp facies. Site 1, Avilion and Thala farms west of the town of Sterkstroom, localities for specimen BP/1/5538 (holotype of *Guttigomphus avilionis* gen. et sp. nov.), along with BP/1/5540, a complete skull in occlusion referred to *Cricodon* cf. *C*. *metabolus*, and the recently described *Impidens hancoxi*. Site 2, Aliwal North, locality of the holotype specimen of *Trirachodon kannemeyeri* (Seeley, 1895a). Site 3, Lady Frere, locality of the holotype specimen of *Trirachodon berryi* (Seeley, 1895a). Site 4, Langberg, Paul Roux district, locality of the holotype of *Langbergia modisei* (Abdala, Hancox & Neveling, 2005). The Burgersdorp outcrop is at its thickest in the area between Lady Frere (Cacadu) and Queenstown (Komani) where it reaches over 600 m. The unit then thins gradually extending from this area to a thickness of just 150–200 m in the area between Aliwal North and Burgersdorp.

Here, we describe one of the specimens (BP/1/5538) previously assigned to *Cricodon metabolus* as a new species, *Guttigomphus avilionis*. This specimen was recovered from the *Cricodon*-*Ufudocyclops* Subzone of the CAZ in the southern exposures of the Burgersdorp Formation, near Sterkstroom (Fig. 2, location 1). We test and discuss its phylogenetic relationships with other gomphodont cynodonts and reconsider the record of *Cricodon metabolus* from South Africa. This new taxonomic record for the *Cricodon*-*Ufudocyclops* Subzone complements the recent description (Tolchard et al., 2021) of another novel trirachodontid (*Impidens hancoxi*) from this subzone, indicating high diversity in the group even in the youngest known strata where they occur.

## Materials and Methods

### Material

**Figure 3:**
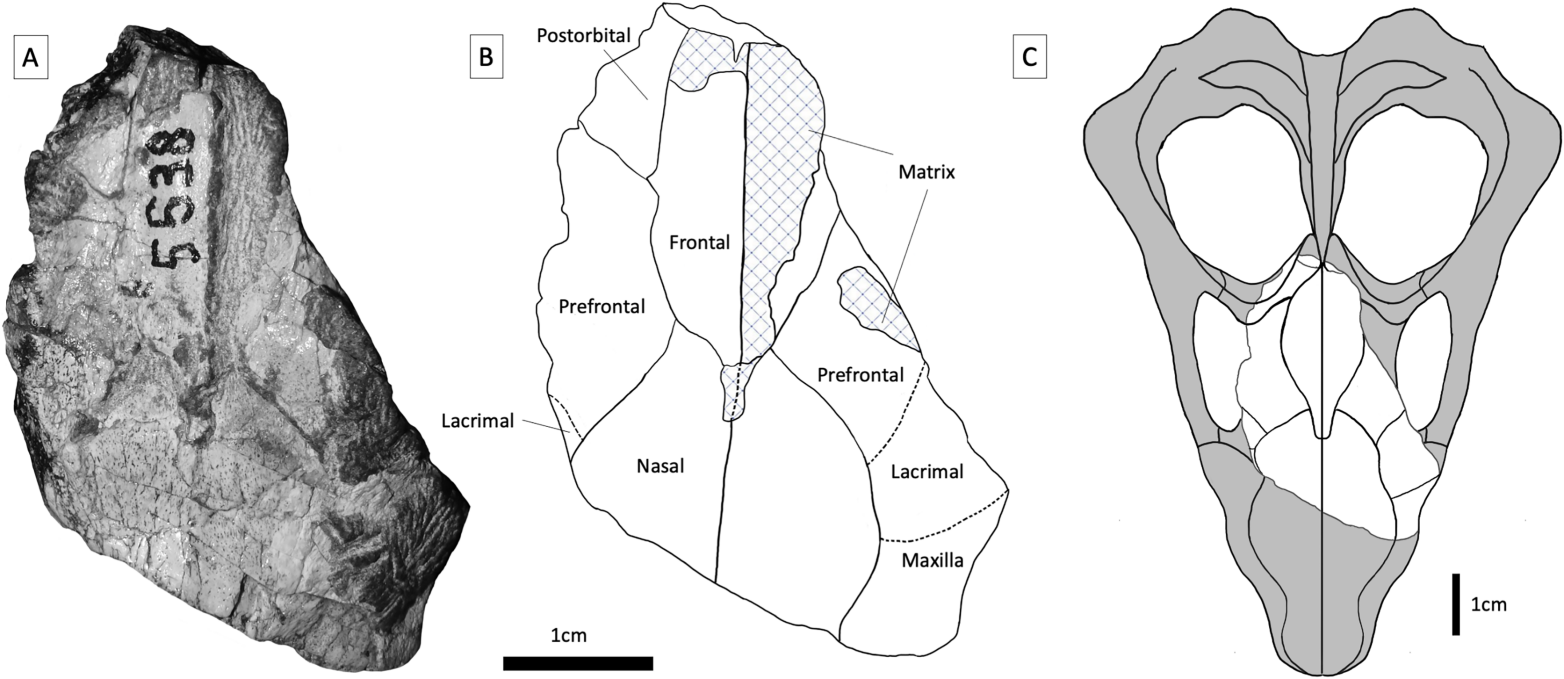
BP/1/5538, holotype of *Guttigomphus avilionis* gen. et sp. nov. (A) Dorsal view. (B) Interpretative drawing. (C) Skull reconstruction showing the part of the skull represented in the specimen in white. The taxa *Langbergia modisei* and *Cricodon metabolus* were used to make informed reconstruction of missing regions of the skull.

**Figure 4:**
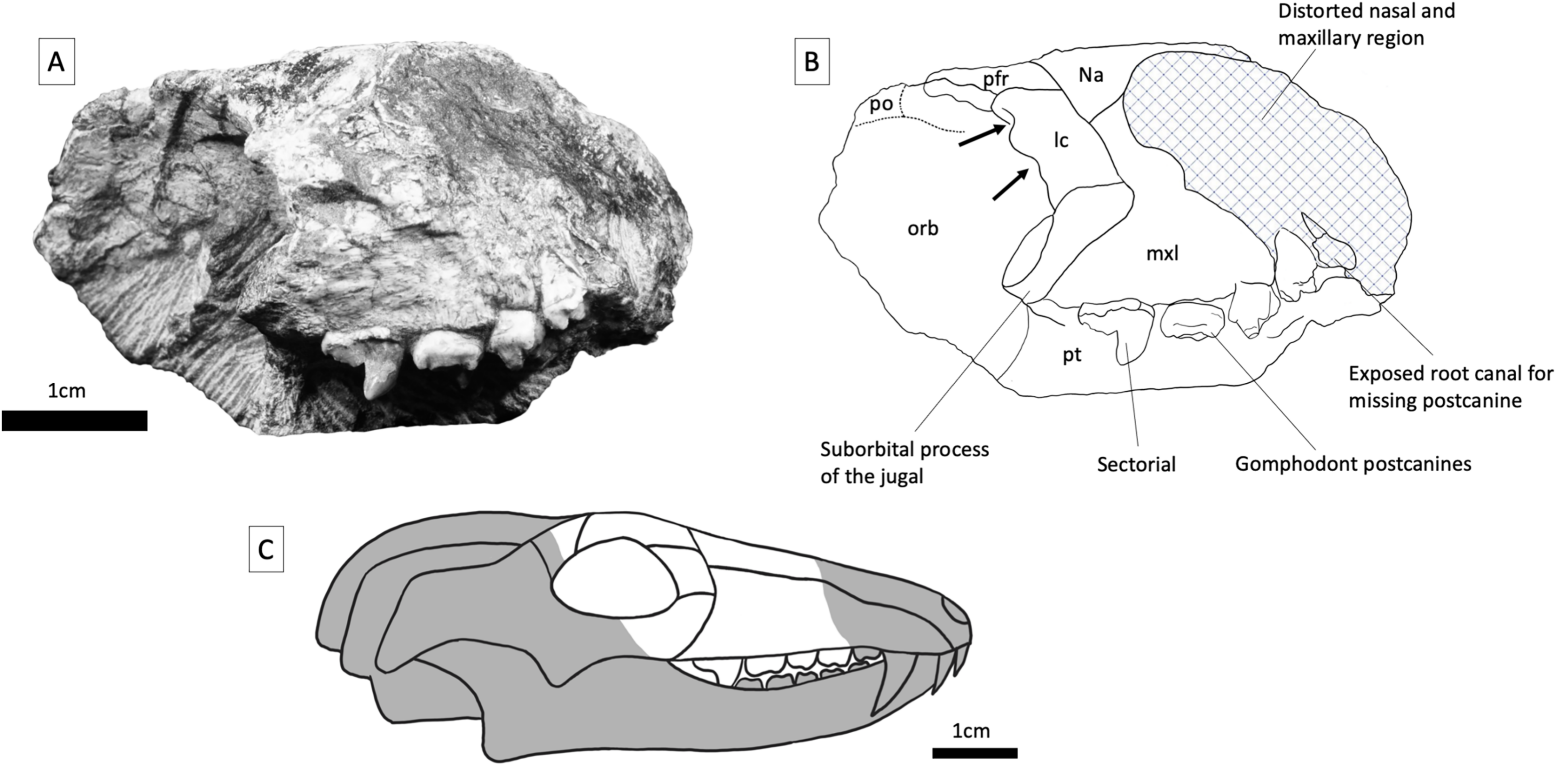
BP/1/5538, holotype of *Guttigomphus avilionis* gen. et sp. nov. (A) Right lateral view. (B) Interpretative drawing. (C) Skull reconstruction showing the part of the skull represented in the specimen in white. Abbreviations: lc, lacrimal; mxl, maxilla; na, nasal; orb, orbital region; pfr, postfrontal; pft, prefrontal; po, postorbital; pt, palatine. Arrows denote location of lacrimal canals.

**Figure 5:**
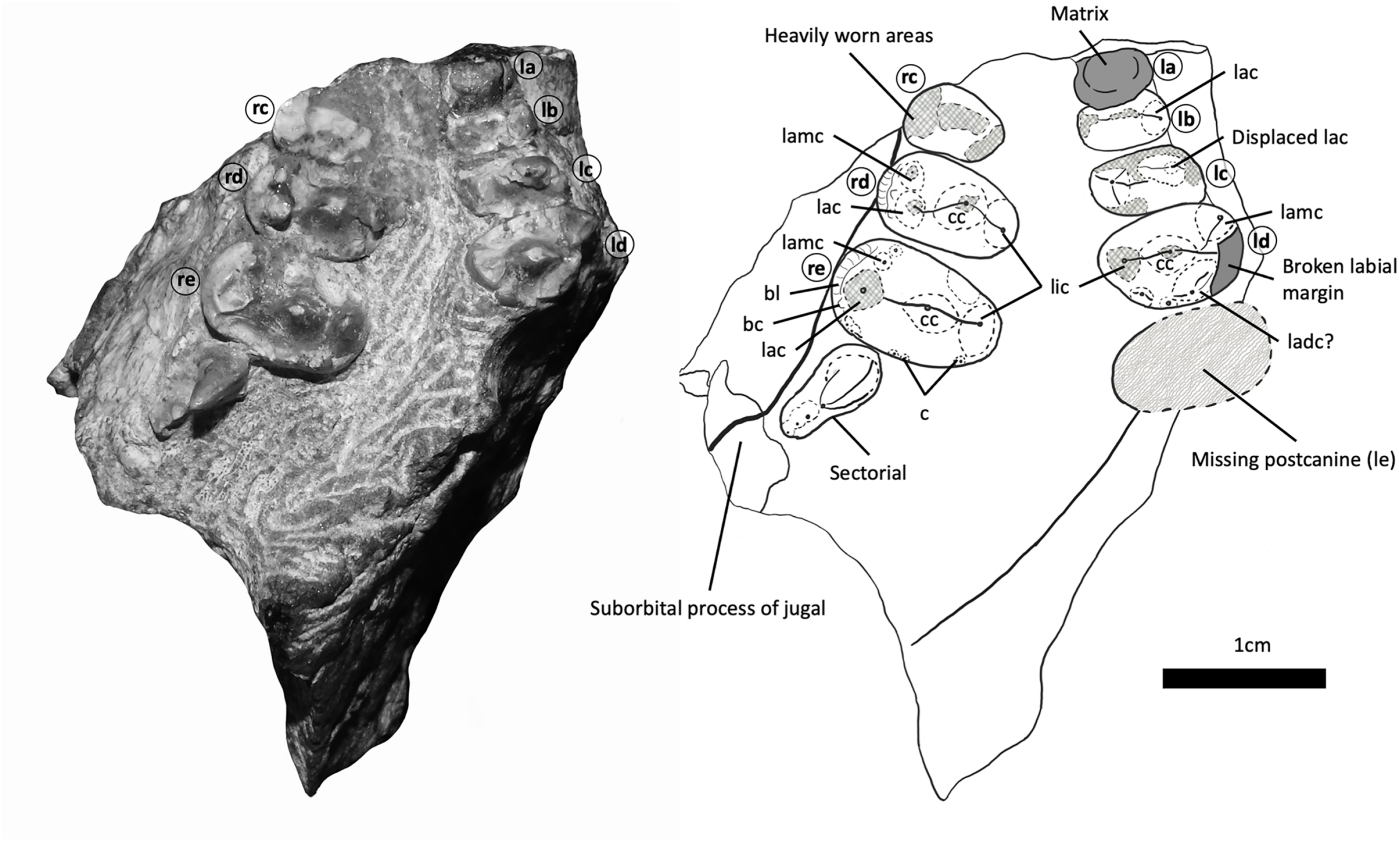
BP/1/5538, holotype of *Guttigomphus avilionis* gen. et sp. nov., in palatal view with interpretive drawing. Teeth have been assigned as Left A (la), Left B (lb), Left C (lc) and Left D (ld), with the alveolus of (missing) Left E (le) and Right C (rc), Right D (rd) and Right E (re). Numerical positional values have not been provided since the number of postcanines in the row cannot be confidently assessed with the material available. Letters have been assigned with the posterior-most gomphodont postcanine being designated as ‘E’ and working forwards across the tooth row. For the purposes of comparative anatomy, the dentitions of other closely related taxa were also characterized in this way. Since the total number of postcanines are subject to intraspecific variation among trirachodontids, the letters assigned in this study will correspond to different numerical loci in different specimens. Abbreviations: bc, buccal cingulum; bl, buccal ledge; c, cuspule; cc, central cusp; lac, labial cusp; ladc, labial distal cusp; lamc, labial mesial cusp; lic, lingual cusp.

Specimen BP/1/5538 was recovered from Avilion Farm, west of the town of Sterkstroom (Fig. 2, location 1), by John Hancox in May 1993. BP/1/5538 consists of a partial skull preserving the posterior portion of the snout and part of the interorbital region (Figs. 3–5). It has undergone transverse and oblique compression such that the right maxillary region has been compressed inwards towards the left orbit. It preserves a large portion of the anterior and dorsal margins of the right orbit, parts of the anterior skull roofing bones, and three reasonably preserved posterior postcanines on each side plus the right sectorial postcanine. BP/1/5538 was previously referred to *Cricodon metabolus* by Abdala, Hancox & Neveling (2005) on the basis of comparative size and tooth morphology, but later excluded from the genus by Hendrickx, Abdala & Choiniere (2019) and recovered outside of Trirachodontidae and inside Traversodontidae in a subsequent phylogenetic analysis (Hendrickx et al., 2020). Hendrickx, Abdala & Choiniere (2019) argued that BP/1/5538 differed from *C*. *metabolus* (and might therefore represent a new taxon) based on the strong apical curvature of the main cusp and two distal accessory cusps on the upper sectorial postcanine (only a single accessory cusp is present in *C*. *metabolus*) and the longer and more ovoid upper gomphodont postcanines with a large labial-mesial accessory cusp and no distal cingulum or ‘step-like’ contact between crowns.

### Methods

For the phylogenetic analysis, a data matrix containing a total of 81 craniodental characters across 24 taxa was assembled, the majority of which were taken from previous studies conducted by Abdala, Neveling & Welman (2006), Kammerer et al. (2008), Gao et al. (2010), and Sidor & Hopson, 2018. To these we added six new characters. Additional characters were: Character 11—absence, presence, or enlargement of the diastema between the upper canines and postcanines, similar to character 3 of Gao et al. (2010) which scored the presence or absence of a diastema between the incisors and canine; Character 12—anterior postcanines are conical, sectorial, less transversely expanded postcanines or the same as more posterior teeth in the row; Character 13—whether the labial or lingual cusp on the upper postcanines has greater sagittal depth; Character 14—whether the wider cusp on the upper postcanines is on the labial or lingual side, or if they are occlusally symmetrical; Character 16—the number of cuspules around the mesial and distal borders of the postcanine crown and Character 17—the percentage difference in the transverse width of upper postcanines in proportion to their length. Scoring of additional characters was conducted based on primary literature as well as photographs of some taxa. The primary objective of our analysis was to determine the relationships of BP/1/5538, so we focused on ingroup relationships within Gomphodontia, the large cynodont clade including Gomphognathidae, Trirachodontidae, and Traversodontidae.

Multiple previous cladistic analyses have recovered *Cynognathus* as the sister taxon of Gomphodontia in the larger clade Cynognathia. Therefore, we used *Cynognathus* as the outgroup for our analysis of gomphodont relationships, as in previous studies (*e.g*., Hopson, 1991; Hopson & Kitching, 2001; Abdala, Neveling & Welman, 2006; Kammerer et al., 2008; Gao et al., 2010; Sidor & Hopson, 2018). Analyses were conducted in TNT version 1.5 for Windows, made freely available with the sponsorship of the Willi Hennig Society (Goloboff & Catalano, 2016), with all characters weighted equally. Fourteen characters were treated as ordered (characters 1, 2, 7, 8, 10, 11, 16, 21, 36, 50, 53, 66, 67, 81). Analyses initially used the default New Technology settings with a driven search, which aims to reduce the number of trees obtained during independent replication (Goloboff & Pol, 2007). The recovered trees were saved to RAM and used as the basis for a traditional search using the tree bisection and reconnection (TBR) swapping algorithm. Eight characters were parsimony uninformative but were retained primarily for the benefit of future studies, as their inclusion in previous matrices across different taxa has been informative. Support values for the results were calculated using bootstrap analysis (1,000 replicates). Synapomorphies were mapped onto the resultant strict consensus of the recovered most parsimonious trees.

The electronic version of this article in Portable Document Format (PDF) will represent a published work according to the International Commission on Zoological Nomenclature (ICZN), and hence the new names contained in the electronic version are effectively published under that Code from the electronic edition alone. This published work and the nomenclatural acts it contains have been registered in ZooBank, the online registration system for the ICZN. The ZooBank LSIDs (Life Science Identifiers) can be resolved, and the associated information viewed through any standard web browser by appending the LSID to the prefix http://zoobank.org/. The LSID for this publication is: 57E697ED-DEC1-4E2D-A2E9-A6EC2E9A0FCA. The online version of this work is archived and available from the following digital repositories: PeerJ, PubMed Central and CLOCKSS.

## Results

### Systematic palaeontology

THERAPSIDA Broom, 1905

CYNODONTIA Owen, 1861

GOMPHODONTIA Seeley, 1895a

TRIRACHODONTIDAE Crompton, 1955

*GUTTIGOMPHUS AVILIONIS* gen. et sp. nov.

**Holotype—**BP/1/5538. A partial skull preserving the posterior snout and right interorbital region.

**Horizon and locality—**BP/1/5538 was recovered from Avilion Farm, west of the town of Sterkstroom in the Karoo Basin of South Africa from the *Cricodon-Ufudocyclops* subzone of the *Cynognathus* Assemblage Zone, Burgersdorp Formation and as such is most likely dated to the early Middle Triassic (Anisian, Hancox et al., 1995).

**Etymology—**From the Latin *gutta*, meaning a fluid drop, and *gomphus*, meaning a peg, referring to the somewhat droplet-like shape (narrower at one end) of the gomphodont postcanines in occlusal view. The species name *avilionis* refers to Avilion Farm, where BP/1/5538 was collected.

**Diagnosis—**A combination of features distinguishes *Guttigomphus avilionis* from other early gomphodonts, including trirachodontids and basal traversodontids such as *Pascualgnathus* and *Etjoia*. The new taxon can be distinguished from *Langbergia modisei* by the absence of distinct, well-developed cingular cuspules on the upper gomphodont postcanines, location of the central cusp closer to the lingual cusp (rather than equidistant from lingual and labial cusps as in *Langbergia*) on the upper gomphodont postcanines, and the absence of a mesial accessory cusp on the sectorial postcanine(s). The new taxon can be distinguished from *Trirachodon* (both species) and *Cricodon metabolus* by the shape of the upper gomphodont postcanines (more ovate, with the lingual margin narrower than the labial margin; the opposite is the case in *Trirachodon* and *Cricodon*), presence of a well-developed labial-mesial accessory cusp on the upper gomphodont postcanines, weaker development of the transverse crest connecting the three main cusps of the upper gomphodont postcanines, and presence of two distal accessory cusps on the upper sectorial postcanine. The new taxon can be distinguished from the newly described *Impidens hancoxi* by the possession of fewer sectorials in the postcanine tooth row (and shorter contribution of the sectorials to the length of the tooth row), upper gomphodont postcanines that are wider labially than those of *Impidens*, in which these teeth are wider lingually (it should be noted that the postcanines are not preserved in *Impidens*; this inference is therefore based on the different outline of the alveolus in occlusal view), more pronounced transverse expansion of the upper postcanines than in *Impidens*, less oblique inclination in the posterior-most postcanines in relation to the axis of the skull than in *Impidens*, and much smaller size than in *Impidens* (which is unlikely to be attributable to immaturity in BP/1/5538, given the observed tooth wear).

Distinction from the traversodontid gomphodonts *Pascualgnathus* and *Etjoia* is predominately on the basis of a wider labial cusp on the upper postcanines (lingual cusp is wider in *Etjoia, Cricodon*, and *Andescynodon*; these cusps are generally symmetrical in other cynognathians) and the number and prominence of cuspules around the crown borders, with *Guttigomphus avilionis* presenting only faint cuspules similar to those found on more derived gomphodonts such as *Scalenodon angustifrons*, whereas *Etjoia* presents a small number of larger cuspules closer to the morphological condition found more commonly in Trirachodontidae.

**Description**

**Maxilla**—Parts of the maxilla are preserved on both sides of the skull (Figs. 3 and 4). The right maxilla has been compressed inwards towards the left orbit, causing distortion to the palate (Fig. 5). The lateral surfaces of both maxillae are badly eroded. The best-preserved portion of the lateral maxillary surface is immediately above the tooth row of the right maxilla, but even here there are visible trabeculae, indicating that this is not intact bone surface. On the left side, the fragmentary section of the left maxilla contains three gomphodont posterior postcanines as well as a fragment of the base of the crown of a preceding postcanine that is heavily obscured by matrix. Also preserved is the anterior part of the alveolus of the missing most posterior gomphodont postcanine (Fig. 5). On the right side, the anterior portion of the right maxilla has been eroded off, but the preserved portion includes the last three gomphodont postcanines and one sectorial postcanine. At the anterior edge of the right maxilla there is part of an additional postcanine, nearly all of which has been sheared off to expose the internal cavity of the root of the tooth. On the right side of the specimen, the suborbital process of the jugal contacts the maxilla above the sectorial tooth, with this suture extending anterodorsally at approximately 45 degrees to the horizontal. The maxillary postcanines are only weakly inset from the lateral maxilla surface, and there is not a distinct labial shelf, but given the amount of distortion and erosion of the maxilla this should not be taken as a definite indication of this structure’s absence in life. The lacrimal contacts the maxilla posterodorsally, also above the sectorial postcanine. Cross sections through the maxillae are exposed anteriorly, where the specimen has been damaged, but provide little useful information. The maxillae are transversely thick in the postcanine region, comparable to the dimensions of other gomphodonts (*e.g*., *Massetognathus*; Crompton et al., 2017).

**Nasal**—Large portions of the posterior parts of the nasals are present (Fig. 3), but their anterior parts have broken away. The dorsal surface of the paired nasals is flat to gently convex transversely. The contact between each nasal and the prefrontal extends from anterolateral to posteromedial at an acute angle, making the posterior processes of the nasals almost equilaterally triangular, which is not typical among gomphodonts. At their posterior ends, the nasals are separated from one another by a triangular anterior projection of the paired frontals.

**Frontal**—The right frontal is almost entirely intact, with only the posterior tip missing, but the left frontal is either missing or almost entirely obscured by overlying matrix. The sutural contact between the frontal and prefrontal runs anteromedial to posterolateral at a weak angle. The frontal margin appears to be convex at its contact with the prefrontal on the right side; by contrast, on the left side this suture is largely straight. This variation may reflect taphonomic distortion or simply natural asymmetry. Although damaged and partially obscured by matrix, a pointed process of each frontal is apparent at its anterior end: together, these processes form a triangular projection between the nasals in dorsal view. Narrow anterior processes of the frontals are frequently present in traversodontids (*e.g*., *Dadadon*; Ranivoharimanana et al., 2011). By contrast, anterior processes of the frontals are usually present but relatively short and broad in trirachodontids (*e.g*., Sidor & Hopson, 2018). The posterior portion of the frontal is missing on each side, but based on the right side the frontal appears to taper towards the midline, flanked by the postorbital, as seen in other trirachodontid specimens (*e.g*., the *Langbergia modisei* skulls NMQR 3255 and NMQR 3280).

**Prefrontal and lacrimal**—The right lacrimal is clearly identifiable due to the presence of paired lacrimal canals on the internal surface of the anterior orbital margin. Lacrimal canals cannot be identified on the left side, as the specimen is broken just before the anterior border of the orbit. The lacrimal contacts the nasal and maxilla anteriorly and the suborbital process of the jugal ventrally. The contact between the lacrimal and the prefrontal is not readily identifiable due to the preservation of the specimen but would have been around the anterodorsal corner of the orbit. Part of the prefrontal is preserved on both sides of the specimen (although the left prefrontal is largely covered in matrix), and this element is trapezoidal in shape, with weakly-angled contacts with the nasal and frontal dorsally. Posteriorly, the prefrontal contacts the postorbital, excluding the frontal from the orbital margin.

**Postorbital**—Only a small fragment of the postorbital is present on the right side. It formed part of the posterodorsal margin of the orbit. A triangular anteromedial postorbital process extends between the frontal and prefrontal in this region.

**Orbit**—The anterior and dorsal margins of the right orbit are preserved, and are formed by the postorbital, prefrontal, lacrimal, and suborbital process of the jugal. There is a large amount of matrix within the inner surface of the orbit, obscuring some of the internal boundaries. On the left side, portions of the lacrimal and prefrontal are present but broken just before the orbital boundary, so that only parts of the inner orbit can be identified.

**Dentition—**Three upper gomphodont postcanine crowns are preserved on each side of the specimen (Fig. 5). Since it is not possible to confidently assess the anterior end of the tooth row, individual teeth have been designated as letters rather than numerical identifiers. In both our description of the new taxon and our comparative anatomy the letter ‘E’ has been assigned to the posteriormost gomphodont postcanine, immediately before the first sectorial, or in taxa where no sectorials are present, to the last tooth in the row. The letters then work sequentially forwards from this point, with teeth A–E representing the five posteriormost gomphodont postcanines. The anteriormost preserved postcanine on the right side (Fig. 5, rc) is heavily worn and only the occlusal outline and a portion of occlusal basin surface area can be seen. The lingual cusp is partially covered by matrix, making accurate measurement difficult, but the transverse width is approximately 5.0 mm and mesiodistal length of the labial cusp is 3.2 mm. The transverse crest was positioned roughly on the midline of the crown but has broken away. The second tooth on the right side (Fig. 5, rd) is better preserved and has two distinct cusps on the labial margin, a primary labial cusp and a smaller but distinct labial-mesial cusp, both of which are missing the apex. The mesiolingual portion of the tooth is slightly obscured by matrix and the main lingual cusp is present but damaged and has undergone slight displacement towards the posterior of the tooth due to taphonomic distortion. The central cusp is missing the apex and is positioned at a slightly oblique angle so that the labial part of the transverse crest is angled towards the distal margin of the tooth and the lingual part is angled towards the mesial margin. The distal border of the crown, where a distal cingulum is usually present in gomphodonts, is heavily worn. The transverse width of tooth rd is 7.2 mm and the wider labial margin is well exposed, measuring 4 mm in mesiodistal length at the widest point compared to the 3.3 mm mesiodistal length of the widest point of the lingual margin.

**Figure 6:**
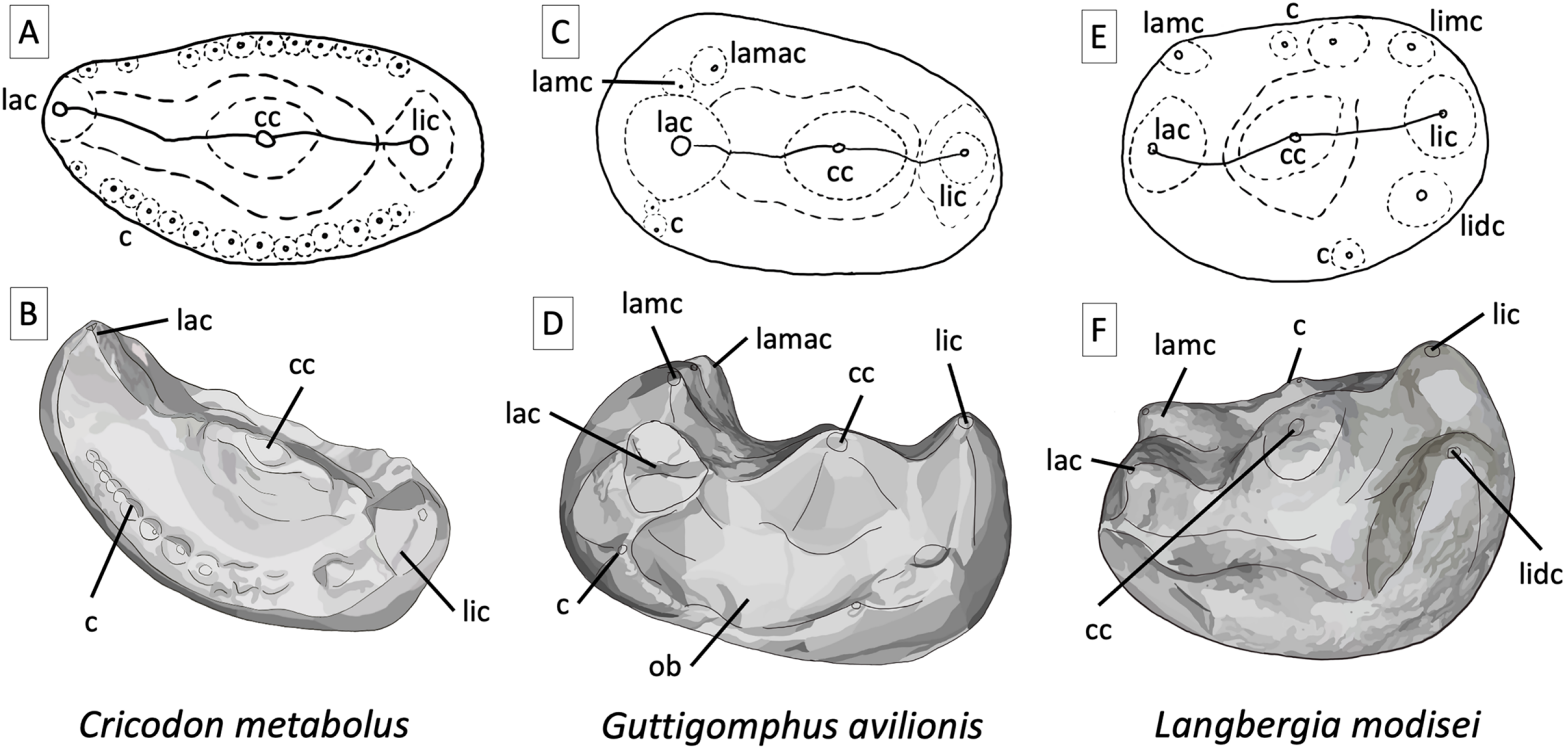
Comparative tooth morphology for trirachodontids, showing the uniquely droplet shaped occlusal outline found in *Guttigomphus avilionis* gen. et sp. nov. and the clear ring of cuspules and more pronounced labial cusp present in *Cricodon metabolus*. (A) *Cricodon metabolus* upper postcanine, interpretive drawing of occlusal outline from UMZC T905; (B) *Cricodon metabolus*, artistic rendition of upper postcanine from UMZC T905; (C) *Guttigomphus avilionis* gen. et sp. nov., interpretative drawing of occlusal outline from BP/1/5538 (re Fig. 5); (D) *Guttigomphus avilionis* gen. et sp. nov., artistic rendition of upper postcanine from BP/1/5538 (tooth re, Fig. 5); (E) *Langbergia modisei* upper postcanine, interpretive drawing of occlusal outline from NMQR 3255; (F) *Langbergia modisei*, artistic rendition of upper postcanine from NMQR 3255. Abbreviations: c, cuspule; cc, central cusp; lac, labial cusp; lamac, labial mesial accessory cusp; lamc, labial mesial cusp; lic, lingual cusp; lidc, lingual distal cusp; limc, lingual mesial cusp; ob, occlusal basin.

**Figure 7:**
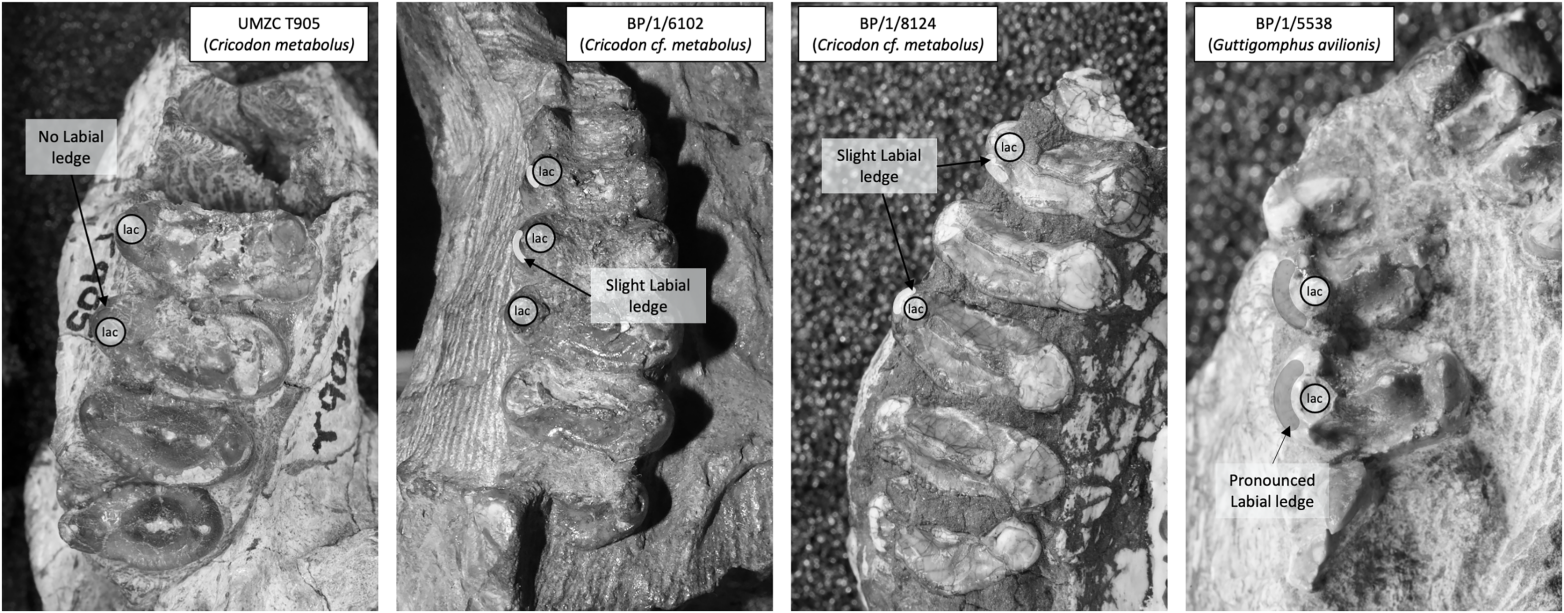
Comparison of the mesial border of the upper postcanines in *Cricodon metabolus* and *Guttigomphus avilionis*. Comparison of the mesial border of the upper postcanines in *Cricodon metabolus* (UMZC T905), two additional specimens of *Cricodon cf. metabolus* from the *Cricodon-Ufudocyclops* subzone (BP/1/6102 and BP/1/8124) and *Guttigomphus avilionis* (BP/1/5538), highlighting the difference in position of the labial cusp. lac, labial cusp.

The posteriormost gomphodont postcanine on the right side (Fig. 5, re; Fig. 6) is the best-preserved member of the series and clearly displays the unique occlusal outline of this taxon, with a transverse width of 8.5 mm, the labial cusp measuring 4.8 mm in mesiodistal length at the widest point, and the lingual base measuring 4.1 mm at the widest point. The main labial cusp has been broken near the base. Lateral to the base of this cusp, a shelf-like expanse of crown separates the cusp from the tooth margin and is interpreted as a cingulum. This contrasts with the condition in *Cricodon metabolus*, where the labial cusp is part of the labial tooth margin and the entire border of the crown slopes gently upwards to an apex that displays slight curvature towards the lingual side (Figs. 6 and 7). A well-developed labial-mesial accessory cusp is present in postcanine re, situated anterior to the transverse midpoint of the main cusp. The labial-mesial cusp is mesiodistally longer than wide. Distal to the main labial cusp, two small cuspules are present at the labial corner of the distal cingulum. These we interpret as members of the series of cuspules usually present along the cingulum in trirachodontids, with the rest lost to wear and/or damage. We agree with the observations of Hendrickx, Abdala & Choiniere (2019), who considered the distal cingulum absent in this specimen, contrary to the original description of Abdala, Hancox & Neveling (2005) that it is present but worn. The relatively small size of the preserved cuspules is similar to that of most trirachodontids and some traversodontids, but differs strongly from that of *Langbergia*, where the cingular cuspules are unusually well-developed. The central and lingual cusps in this tooth are well preserved, the transverse crest is straight, running between the lingual and labial cusps and bisecting the central cusp; without the oblique angle observed in the preceding tooth (tooth rd). The central cusp is positioned closer to the lingual than the labial margin, and there are deep occlusal basins to either side forming a concave surface between the cingula and the main cusps. The labial borders of teeth rd and re have a very gently sloping, almost flat ledge, 1–2 mm across, positioned labial to the main labial cusp. On the lingual side, the base of the main cusp merges into the border of the crown with no separating ledge.

The sectorial postcanine on the right side is well preserved but incompletely erupted, and measures 2.2 mm wide transversely and 6 mm long mesiodistally. The main cusp displays a posterior curvature towards the apex (comparative to other gomphodonts) and is very faintly serrated (difficult to observe with the naked eye). At least two distal accessory cusps (similar to those of *Langbergia*) are present on the sectorial tooth, but their apices are broken. Consistent with the rest of the post-canine series, the sectorial also possesses a sectorial labial ledge, a feature commented on by Hendrickx et al. (2020) who suggest it might result from ‘lingomesial rotation of the broken crown’. Given the presence of a ledge on the gomphodont postcanines (Fig. 7) it is possible this is a genuine anatomical feature.

On the left side, the anterior-most tooth position (Fig. 5, la) is largely obscured by matrix and only a tiny portion of the labial margin of a tooth can be seen in lateral view. Assuming that its dimensions are accurately reflected by the overlying chunk of sediment, the crown is approximately 3.5 mm in transverse width. The second most anterior tooth on the left side (Fig. 5, lb) is heavily eroded and partially overlaid by matrix, preventing accurate measurement of the lingual cusp; however, the difference between the mesiodistal lengths of the lingual cusp and labial cusp appears less pronounced on tooth lb than the other postcanines in the series. The labial cusp is mostly preserved, missing the apex and cracked at the base, indicating that minor taphonomic displacement may have occurred. The central ridge sits at a slightly oblique angle as seen in tooth rd, so that the lingual side of the ridge is positioned towards the mesial border of the crown and the labial side of the ridge meets the midline. Tooth lb measures 4.6 mm in transverse width. The subsequent tooth (Fig. 5, tooth lc) shows substantial damage to the mesial and distal borders of the crown. The lingual cusp is only partially preserved, and the labial cusp has been displaced and sits over the central cusp with a small portion of the labial border of the crown missing. Teeth lb and lc both have the transverse ridge positioned fractionally more towards the anterior border of the crown. Tooth lc measures 5.6 mm in transverse width and 2.4 mm in mesiodistal length at the base of the lingual cusp. The labial cusp cannot be accurately measured due to damage.

The posteriormost tooth on the left side (Fig. 5, ld) has sustained breakage of the labial side so that a large portion of the tooth root, including some of the internal cavity, has been exposed laterally on the specimen. Only the base of the lingual cusp is preserved, along with an intact smaller distal lingual accessory cusp (which also appears to bound the edge of the distal cingulum) and faint distal cuspules. The central cusp is well preserved, missing only the apex. It exhibits the slight distortion of the angle of the transverse ridge with the lingual side being angled towards the mesial margin and the labial side being angled towards the missing labial cusp. This angling of the transverse ridge is also observed in *Trirachodon berryi* (BP/1/4658). There is a labial-mesial cusp and a large feature which is possibly a labial distal cusp, but since the distolabial portion of the tooth is missing it is not possibly to accurately gauge this feature in relation to the labial cusp or crown borders. Tooth ld measures 6.2 mm in transverse width and 2.9 mm in mesiodistal length across the base of the lingual cusp. Measurement of the labial cusp is not possible due to damage, but the widening trajectory of the mesial and distal crown borders clearly display the hallmark widening of the tooth on the labial side forming the droplet shaped occlusal outline.

The morphology of the upper postcanine teeth provides the primary evidence for recognition of *Guttigomphus avilionis* as a new species. The overall morphology appears somewhat intermediate between *Langbergia modisei* and *Cricodon metabolus* (Fig. 6). The shared features which typify trirachodontids, as first outlined by Crompton (1955), are that they all display transverse expansion of the postcanines, three cusps in a transverse row across the upper postcanines, and cuspules around the crown borders. *Guttigomphus* shares with *Langbergia* a distinct labial-mesial accessory cusp, which is absent in *Cricodon* and *Trirachodon*. Transverse expansion of the crowns in *Cricodon, Langbergia* and *Guttigomphus* is less than in *Trirachodon berryi* and *T*. *kannemeyeri*, with the labiolingual width being typically 75–95% greater than the mesiodistal length, compared to a labiolingual width in excess of twice the mesiodistal length in *Trirachodon* (Table 1). Details of the occlusal surfaces further differ between *Guttigomphus* and *Cricodon*/*Trirachodon*, with the latter having a broader lingual margin and the former having a broader labial margin. There are also often distinct minor accessory cusps on the labial and lingual sides that are more prominent than the crown border cuspules in *Cricodon* and *Trirachodon*, which appear as small spherical ‘bumps’. In *Guttigomphus*, these smaller accessory cusps rise with the sagittal (dorsal:ventral) plane in line with the border of the crown and form individual pointed apices, rather than appearing as rounded relief structures on the occlusal surface of the crown (Fig. 6).

**Table 1:** Comparative measurements of transverse width and mesiodistal length in upper postcanines across all sampled trirachodontid species.

	*Cricodon metabolus* (NHCC LB28)	*Cricodon metabolus* (UMZC T905)	*Trirachodon kannemeyeri* (SAM-PK-K12168)	*Trirachodon kannemeyeri* (SAM-PK-K171)	*Trirachodon berryi* (NHMUK PV R3579)	*Langbergia modesei* (NMQR 3255)	*Guttigomphus avilionis* (BP/1/5538)
Approximate skull length	180 mm	170 mm	115 mm	100 mm	120 mm	160 mm	170 mm
Approximate length postcanine row	50 mm	55 mm	30 mm	30 mm	30 mm	40 mm	47 mm
Upper PCb width	5 mm	12.5 mm	6.2 mm	7.6 mm	5.5 mm	7.5 mm	8.4 mm
Upper PCb length	3 mm	6.5 mm	3 mm	3.5 mm	2.4 mm	3.7 mm	5.1 mm
% difference	66.7	92.3	106.6	117.7	131	102.7	64.7
Upper PCc width	6.75 mm	13 mm	6.4 mm	7.8 mm	4.7 mm	6.5 mm	6.5 mm
Upper PCc length	3.4 mm	6 mm	2.4 mm	3.7 mm	3.2 mm	3.5 mm	3.9 mm
% difference	98.52	116.6	166.7	166.7	46.8	85.7	66.7
Upper PCd width	7.4 mm	11 mm	4.7 mm	7 mm	4.7 mm	6 mm	6 mm
Upper PCd length	3.75 mm	6 mm	2.5 mm	2.8 mm	2.4 mm	3.2 mm	3.5 mm
% difference	97.30	83.3	88	150	95	87.5	71.4
Upper PCe width	4.2 mm	10 mm	3.75 mm	6.3 mm	6.2 mm	5.2 mm	5 mm
Upper PCe length	3.2 mm	5.5 mm	2 mm	2.7 mm	2.4 mm	3 mm	2.5 mm
% difference	31.25	81.81	87.5	133.3	158	73.3	100
Upper PC average width	5.8 mm	11.6 mm	5.3 mm	7.2 mm	5.5 mm	6.3 mm	6.5 mm
Upper PC average length	3.3 mm	6 mm	2.5 mm	3.2 mm	2.6 mm	3.3 mm	3.7 mm
Average % difference	80.1	93.6	112.9	126.1	120	46.8	75.6

**Note:**

Tooth ‘E’ represents the posterior most gomphodont postcanine, immediately prior to the sectorials. This sequence then runs forwards across the tooth row, teeth A–E are the five most posterior gomphodont postcanines (see Fig. 5). Comparison has been made in this way due to the entire tooth row not being present in all taxa examined, including *Guttigomphus avilionis* gen. et sp. nov. It should be noted that this means ‘tooth E’ will have a different numerical locus identifier across different taxa, as the total number of postcanines is subject to intraspecific variation.

**Figure 8:**
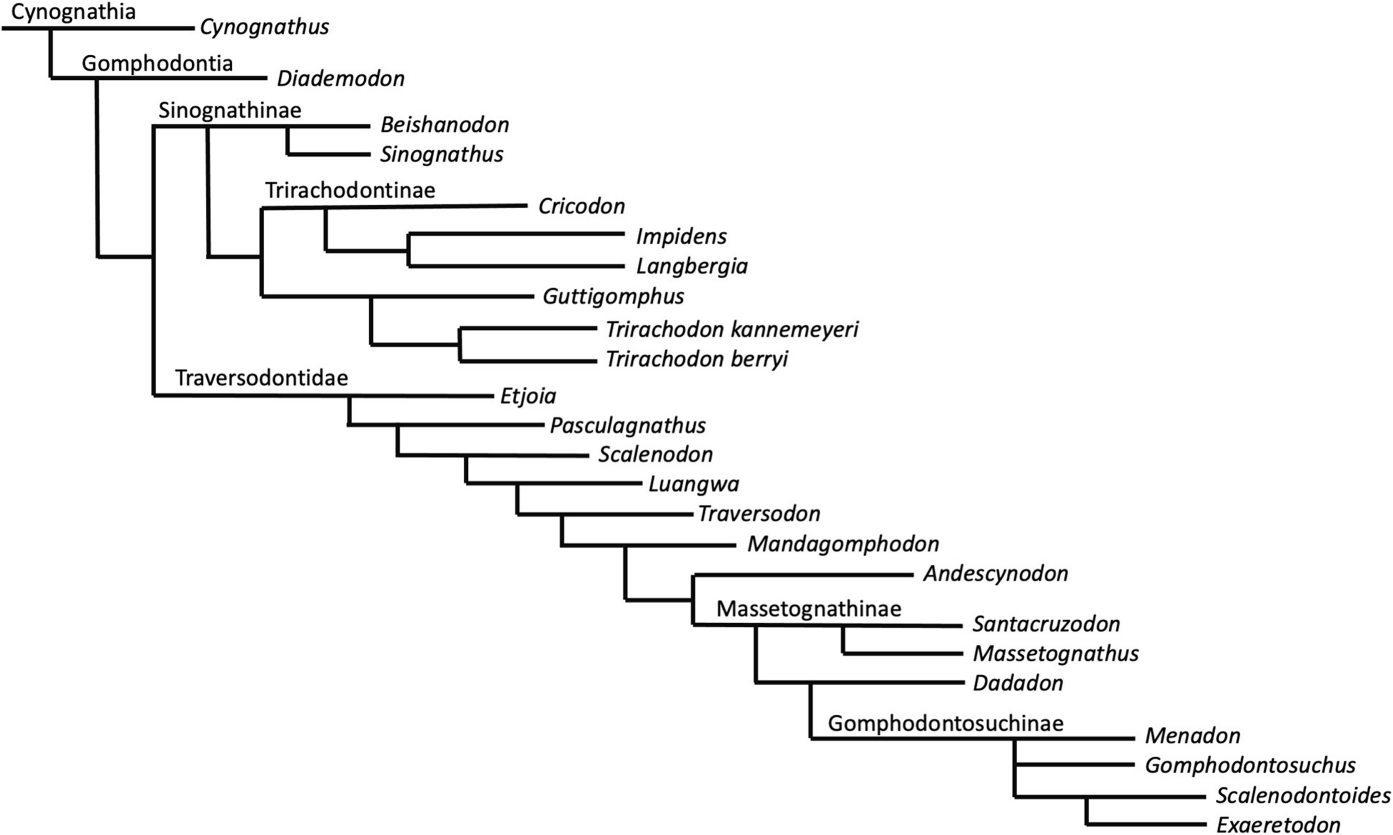
Strict consensus of two equally most parsimonious trees recovered by the phylogenetic analysis showing relationships among gomophodont cynodonts.

**Figure 9:**
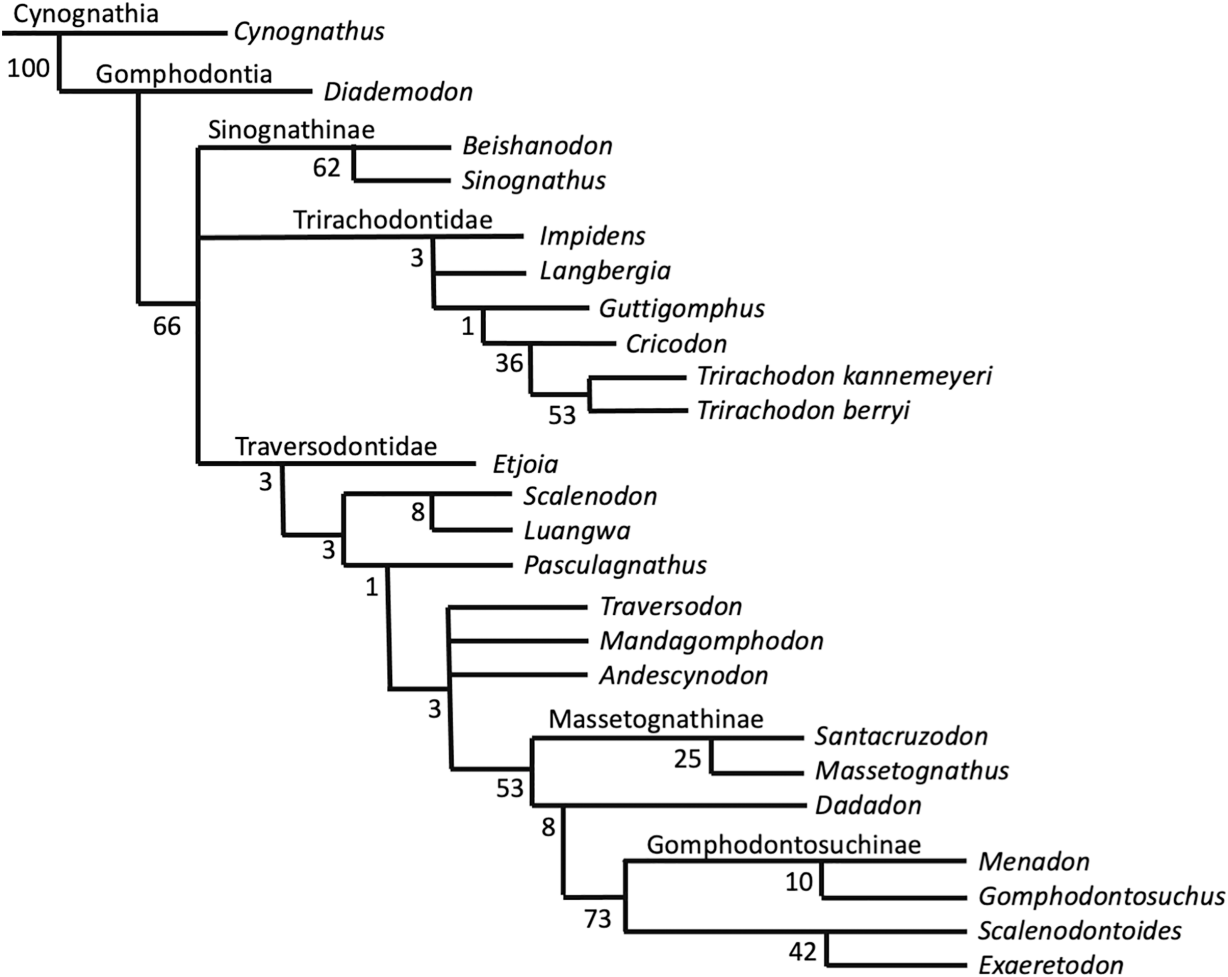
Bootstrap support values for phylogenetic results shown in Fig. 8.

**Phylogenetic results—**The New Technology search in TNT recovered two most parsimonious trees (MPTs) of 233 steps. Subsequently, a traditional search with TBR did not recover any further MPTs. The strict consensus of these two MPTs is displayed in Fig. 8. Bootstrap support is generally low, with a bootstrap value of only 1% for the placement of *Guttigomphus* as the sister taxon to *Cricodon* + *Trirachodon* (Fig. 9), and 62% support for Sinognathinae. The relationship between *Cricodon* and *Trirachodon* has a support value of 36% and the support value for *T. kannemeyeri* and *T. berryi* as sister taxa is 53% (Fig. 9).

*Guttigomphus avilionis* is characterised by the following local autapomorphies in this analysis:

1. Character 14; character state 2, upper postcanine widest on labial cusp.
2. Character 17; character state 1, cuspules around mesial and distal border of upper postcanine crowns are very faint.
3. Character 25; character state 1, distal cingulum on the upper postcanine absent (Abdala, Neveling & Welman, 2006)
4. Character 26; character state 1, upper postcanine labial cingulum absent (Sidor & Hopson, 2018)
5. Character 28; character state 1, deep occlusal basins present (Abdala, Neveling & Welman, 2006)

Our phylogenetic results largely support the results of previous phylogenetic analyses (Gao et al., 2010; Abdala, Neveling & Welman, 2006; Kammerer et al., 2008; Sidor & Hopson, 2018). The only notable differences are that our results do not resolve *Trirachodon kannemeyeri* within the genus *Cricodon*, as suggested by Sidor & Hopson (2018). It has been suggested by other researchers that *Trirachodon kannemeyeri* does not constitute a valid taxon and is instead an ontogenetic variation of *Trirachodon berryi* (Hendrickx, Abdala & Choiniere, 2019). As such, we do not here use the generic recombination of *Cricodon kannemeyeri* proposed by Sidor & Hopson (2018). We retain *T. kannemeyeri* in *Trirachodon* based on historical usage and recognize that additional work is required to address trirachodontid alpha taxonomy. In addition, we do not resolve *Guttigomphus avilionis* within Traversodontidae, unlike the results of Hendrickx et al. (2020). However, we note that these phylogenetic positions are unstable and that morphological similarities, such as the presence of cuspules around the borders of the postcanines, exist between the basal members of both Trirachodontidae and Traversodontidae.

Trirachodontidae can be considered as a monophyletic group of gomphodont cynodonts that now numbers six species and is currently found exclusively in the *Cynognathus* AZ of South Africa and correlated faunal assemblages in Tanzania and Zambia (if the disputed Chinese taxa *Sinognathus* and *Beishanodon* are not included within the clade, see discussion).

Synapomorphy mapping in TNT reconstructs the following character state transformations as unambiguous synapomorphies of Trirachodontidae:

1. Character 12: character state 2, the anterior most postcanines on the maxillary tooth row are less transversely expanded post canines (rather than conical or sectorial).
2. Character 17: character state 2, a multitude of tiny cuspules present on the mesial and distal upper postcanine crown border.
3. Character 18: character state 2, the upper postcanine row consists of gomphodont and sectorial postcanines (conical postcanines absent).
4. Character 40: character state 2, three cusps on the transverse row of the lower postcanines.

## Discussion

The discovery of *Guttigomphus avilionis* adds to our expanding knowledge of cynodont diversity in the *Cynognathus* Assemblage Zone. There are now at least nine cynodont taxa reported from probable Middle Triassic beds in the Karoo Basin of South Africa: *Diademodon tetragonus* (Kitching, 1958; Brink, 1963), *Cynognathus crateronotus* (Brink, 1963), *Lumkuia fuzzi* (Hopson & Kitching, 2001), *Bolotridon frerensis* (Seeley, 1895b; Pusch, Kammerer & Fröbisch, 2021), *Trirachodon berryi* (Seeley, 1895a; Huene, 1911), *Trirachodon kannemeyeri* (Seeley, 1895a; Colbert & Kitching, 1977), *Cricodon metabolus* (Crompton, 1955; Abdala, Hancox & Neveling, 2005), *Impidens hancoxi* (Tolchard et al., 2021) and now *Guttigomphus avilionis* (the poorly known *Cistecynodon parvus* may represent a tenth, but requires restudy to confirm its validity). Of these, the specimen that we identify here as *Guttigomphus avilionis* was one of a number of specimens from CAZ Subzone C previously referred to *Cricodon metabolus*.

*Guttigomphus* falls out as the earliest branching member of Trirachodontidae. It shares some features in common with the recently described early traversodontid *Etjoia*, such as the extent of transverse expansion on the upper post-canines, the presence of a mesial cingulum and the presence of deep occlusal basins. These features are absent in more derived trirachodontids but commonplace within Traversodontidae. These two taxa reflect the symplesiomorphies of the common ancestor between trirachodontids and traversodontids.

### Discussion of trirachodontid material from the *Cricodon*-*Ufudocyclops* Subzone

Specimen BP/1/5538 (*Guttigomphus avilionis)* was recovered in May 1993 by John Hancox, from the same general location as specimens BP/1/5540, BP/1/5835, and BP/1/6102. All of these were previously referred to *Cricodon metabolus* by Abdala, Hancox & Neveling (2005) and then ‘tentatively referred to *Cricodon’* by Hendrickx, Abdala & Choiniere (2019). In addition to these specimens, collaborative fieldwork in 2014 between the University of Birmingham and the University of the Witwatersrand recovered an additional trirachodontid specimen: BP/1/8124. A full evaluation of the taxonomic status of all these specimens is the subject of ongoing work, and we provide only some brief comments here.

**Figure 10:**
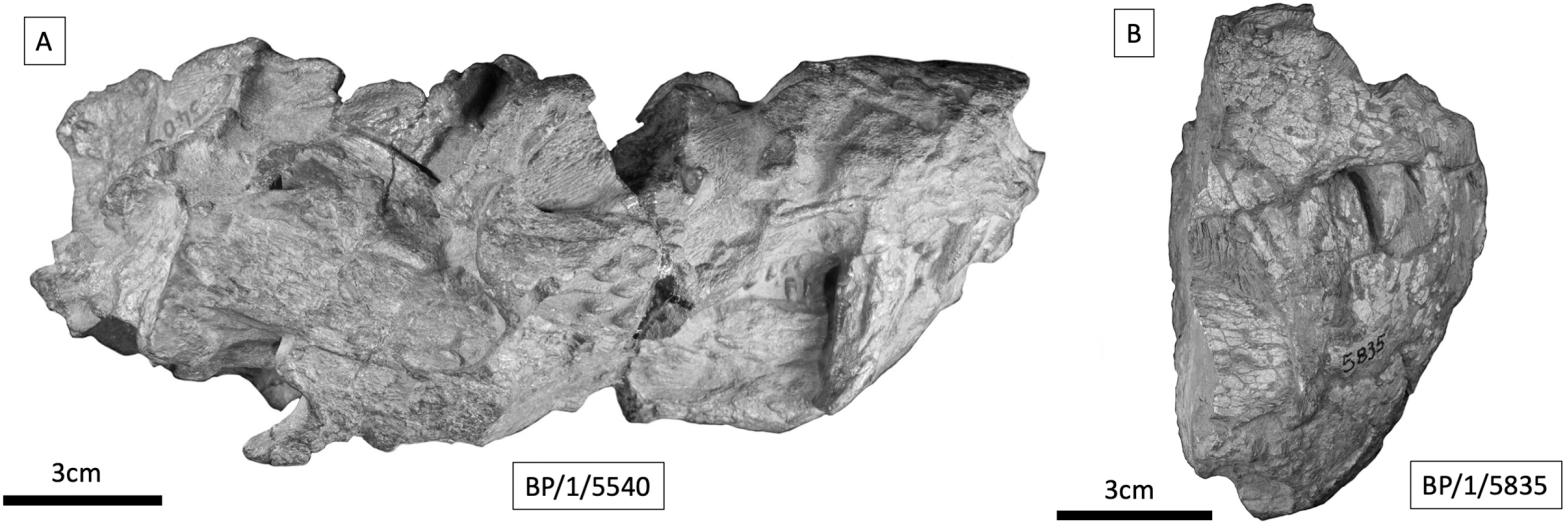
Cynodont specimens from the *Cricodon-Ufudocyclops* subzone. (A) BP/1/5540, *Cricodon* cf. *C. metabolus*. Full skull in occlusion, right lateral view. (B) BP/1/5835, large cynognathian, probably *Cynognathus*, right lateral view of anterior part of skull.

BP/1/5540 is a complete but poorly preserved skull with the lower jaws in occlusion, measuring 180 mm from the anterior of the snout to the posterior of cranium (Fig. 10A), and is comparable in size to the *Cricodon metabolus* holotype. It is not possible to confidently identify BP/1/5540 as *Cricodon* without examination of postcanine morphology. Due to the iron-heavy composition of the matrix, CT data have proved uninformative to date. Based upon external morphology, nothing was identified to exclude BP/1/5540 from *Cricodon metabolus*. If BP/1/5540 does prove to be an additional example of *Cricodon metabolus*, it provides additional morphological information on phylogenetic characters that were scored as unknown on the holotype, given that the preservation of the dentary, palate, and some other skull elements are good. BP/1/5540 provides information on the greatest width of the zygomatic arch, the length of the snout in proportion to the temporal region, and the widest part of the temporal fenestra, which are uncertain in the type material. At present, however, we support previous researchers in only tentative assignment to *C. metabolus*.

BP/1/5835 is an anterior portion of snout broken just posterior to the canines (Fig. 10B). In addition to this there are two fragments of upper and lower jaw in occlusion (Abdala, Hancox & Neveling, 2005), although this additional material was not available for examination for the purposes of this article. Estimating the skull length with such fragmentary material is more challenging, but it appears to represent a larger individual with a total skull length of up to 230 mm. The canine appears proportionately smaller than in BP/1/5540. BP/1/5835 and BP/1/5540 differ in the presence of procumbent incisors in the latter. BP/1/5540 also possesses canines and incisors with proportionately greater sagittal (dorsal:ventral) depth relative to the basal width than BP/1/5835 where the incisors and canines appear less slender (Fig. 10). The additional material indicates the sequence of the postcanine row does not provide information on distinct gomphodont postcanine morphology without CT data. Based on its size and the morphological differences observed in the incisors, it seems unlikely that BP/1/5835 is a trirachodontid. Firm conclusions cannot be drawn from such fragmentary material, but based on the larger size, lack of incisor procumbency, and proportionately stouter mesial:distal depth to basal width in the canines and incisors, it seems more consistent with basal members of Cynognathia, such as *Cynognathus* or possibly *Diademodon*. Further examination of this specimen, particularly through obtaining informative CT data of the postcanine morphology, is required in the future.

**Figure 11:**
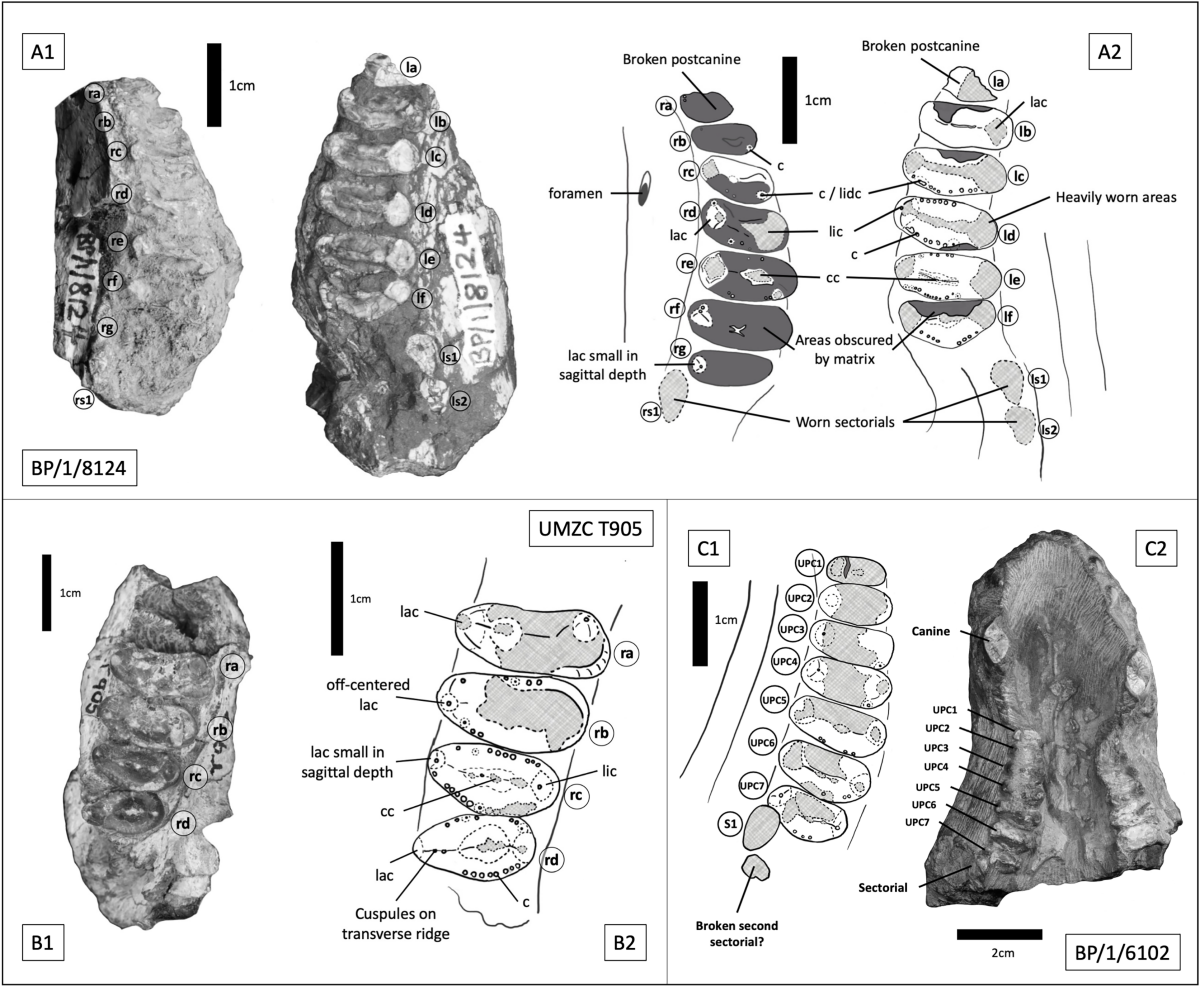
Trirachodontid specimens from the *Cricodon-Ufudocyclops* subzone of South Africa and the Manda Beds of Tanzania. (A1) BP/1/8124, *Cricodon* cf. *C. metabolus*, *Cricodon-Ufudocyclops* subzone, South Africa. Left and right fragments of maxillary cheek teeth row, occlusal view. (A2) Interpretive drawing of BP/1/8124, occlusal view. (B1) UMZC T905, *Cricodon metabolus* holotype, Manda Beds, Tanzania. (B2) Interpretive drawing of UMZC T905. (C1) BP/1/6102, *Cricodon* cf. *C. metabolus*, *Cricodon-Ufudocyclops* subzone, South Africa. Interpretative drawing in occlusal view. (C2) BP/1/6102, photograph in occlusal view. Abbreviations: c, cuspule; cc, central cusp; lac, labial cusp; lic, lingual cusp; lidc, lingual distal cusp; 1s1, left sectorial one; ls2, left sectorial two; rs1, right sectorial one. Teeth have been assigned as Left A (la), Left B (lb), Left C (lc) and Left D (ld) and Right C (rc), Right D (rd) and Right E (re) to distinguish left from right (see caption to Fig. 5). Numerical values have not been provided since the number of postcanines in the row cannot be confidently assessed with the material available.

BP/1/8124 consists of two maxillary tooth row fragments, from opposite sides of what is inferred to be a single skull, with the dentition of the right side being heavily obscured by matrix. On the right side there are seven gomphodont postcanines and a single sectorial preserved, whereas on the left there are six gomphodont postcanines and two sectorials (Figs. 11 A, 11B). The estimated skull length for BP/1/8124 is approximately 180 mm. BP/1/6102 consists of a large portion of snout, with maxilla, premaxilla and nasals present (Fig. 11 C). Its estimated skull length is identical to that for BP/1/8124. Measurements for BP/1/8124 and BP/1/6102 use specimen BP/1/5540 as a proxy. Both BP/1/6102 and BP/1/5540 measure 42 mm from front of premaxilla to the back of the caniniform. In BP/1/8124 the extend of the postcanine row is comparable to the other two specimens. Four incisors on each side remain well preserved, as are the basal portions of both canines, seven gomphodont postcanines on the right side plus one clear sectorial and an additional fragment which may represent a second sectorial. The left side is less well preserved and contains six heavily worn gomphodont postcanines and the alveolus for the seventh, posteriormost postcanine. Based on their similarity in size, near identical occlusal outline of gomphodont postcanines and in-stepping arrangement of the tooth row (teeth at the anterior most end are smaller and less transversely expanded, there is a curvature of the tooth row towards the lingual side in the middle of the row and becomes more labially offset towards the posterior end of the row) it can be determined that BP/1/8124 and BP/1/6102 represent two specimens of the same taxon. Based on the seven postcanines of BP/1/6102 and the smaller size of the anterior most postcanine on BP/1/8124, it seems probable that the right side of BP/1/8124 represents the entire maxillary tooth row and that both specimens present a total of seven gomphodont postcanines and two sectorials.

Sidor & Hopson (2018) identified paired foramina positioned above the 4^th^ to 5^th^ and 5^th^ to 6^th^ upper postcanines as a diagnostic feature of *Cricodon metabolus*. A single foramen can be identified on the left side of BP/1/8124 above the 3^rd^ to 4^th^ postcanine and on the right side of BP/1/6102 above the 3^rd^ to 4^th^ postcanine. Other features of the postcanines also suggest BP/1/8124 is referable to *Cricodon metabolus*, including the ring of distinctly spherical cuspules found across mesial and distal borders of the crown and a distinct transverse crest which runs from labial to lingual cusps. Labial and lingual borders of the crown present a singular main cusp with no prominent accessory cusps (although teeth rd and rf on BP/1/8124 and tooth rb on UMZC T905 all present a ‘labiomesial cuspule’ which sits more prominently than the other border cuspules immediately to the anterior of the main labial cusp) and a comparable level of gomphodont transverse expansion (Figs. 11A2, 11B2). The *Cricodon metabolus* holotype (UMZC T905) and specimen BP/1/8124 both display a labial cusp small in sagittal depth, appearing more like an accessory cusp or large cuspule than the prominent labial cusp on other teeth (Fig. 11A2, tooth rg; Fig. 11B2, tooth rc).

It is worth noting that *Cricodon metabolus* presents labial cusps that are much narrower at the base than the lingual cusp and the labial cusp itself rises with the labial margin of the tooth, presenting no ledge or cingulum to the labial side of the labial cusp (Figs. 11B1, 11B2). Both BP/1/8124 and BP/1/6102 lack the distinction of the narrower labial cusp and have labial and lingual cusps that are approximately symmetrical in basal width. BP/1/8124, BP/1/6102 and *Cricodon* all have central cusps which sit closer to the lingual border, but in BP/1/8124 and BP/1/6102 they are slightly closer to the centre. The poor state of preservation of these specimens makes confident assessment of the labial margin challenging, but BP/1/8124 and BP/1/6102 also appear to have the labial cusp sitting fractionally in from the labial margin as seen in other trirachodontids (Fig. 7), unlike in the holotype and referred specimens of *Cricodon metabolus*. The anterior upper postcanines in the referred specimen of *Cricodon metabolus* from Zambia (NHCC LB28, Sidor & Hopson (2018) are tricuspid teeth with the cusps arranged mesiodistally, and they resemble sectorials in lateral view. In BP/1/6102, the anteriormost teeth in the row are transversely expanded gomphodont postcanines. In addition, NHCC LB28 presents a total of 10–11 gomphodont upper postcanines rather than the 7 found in BP/1/8124 and BP/1/6102. These differences could be ontogenetic, although it is worth noting that BP/1/8124 and BP/1/6102 are the larger specimens and are unlikely to be juveniles. It is not unheard of for this sort of ontogenetic variation to occur in gomphodonts; in *Exaeretodon* the juveniles have a greater number of postcanines (Abdala, Barberena & Dornelles, 2002). However, juveniles with a greater number of postcanines would be unusual compared to the growth series observed in *Trirachodon* (Hopson, 2005), therefore we must consider the possibility that this difference presents evidence that BP/1/8124 and BP/1/6102 might represent a novel species of *Cricodon*. As such, we currently consider BP/1/5540, BP/1/8124 and BP/1/6102 to represent *Cricodon* cf. *C*. *metabolus* and exclude BP/1/5835, instead proposing that the latter specimen is more likely *Cynognathus*.

### Discussion of disputed trirachodontid taxa

The Chinese taxa *Sinognathus gracilis* (Young, 1959) and *Beishanodon youngi* (Gao et al., 2010) have been difficult to place phylogenetically, and their classification as trirachodontids has been disputed in different cladistic analyses. In their original description of *Beishanodon youngi*, Gao et al. (2010) conducted an analysis of 43 craniodental characters across 18 taxa and found some support in their majority rule consensus tree for the grouping of Sinognathinae and Trirachodontidae. Liu & Abdala (2014) conducted a large-scale phylogenetic analysis across 30 taxa to resolve the interrelationships of Transversodontidae using 18 cranial characters, 32 dental morphology characters, 16 characters pertaining to dental positioning, and 10 postcranial characters, and resolved *Sinognathus* and *Beishanodon* as closest to *Langbergia*. An identical taxonomic resolution was also found by Gaetano & Abdala (2015). However, Sidor & Hopson (2018) argued that Sinognathinae do not belong within trirachodontids, recovering them as traversodontids in their phylogenetic analysis. They speculated that they may instead be probainognathians that have convergently evolved transverse expansion of the postcanines. They identified several morphological features in the Chinese taxa that they considered to support a placement as traversodontids, such as the absence of the parietal foramen and jugal process and the significantly bowed zygomatic arch (Sidor & Hopson, 2018). Another analysis by Hendrickx et al. (2020) recovered *Cricodon* alongside Sinognathinae and suggested that Trirachodontidae is a paraphyletic grouping at the base of Traversodontidae.

Our analysis attempted to include all potentially parsimony informative traits among Cynognathia and the only characters that we excluded that were included in the matrix of Sidor & Hopson (2018) were those that identified differences between more basal taxa (*Procynosuchus, Galesaurus, Thrinaxodon*) and Probainognathia; for example the absence of the prefrontal (found in Tritylodontidae, *Pachygenelus*, and *Morganucodon*) and postorbital, shape of the internarial vomer, the secondary palatal plate not reaching the midline, length of secondary palate relative to the tooth row (shorter in all of Cynognathia) and the dorsal extent of the zygomatic arch. We also excluded characters for postcranial material due to insufficient availability of material for scoring in either the Chinese taxa or our newly described species.

We do not clearly resolve these Chinese taxa as being either trirachodontids or traversodontids. Although recovered as the sister taxon of the African trirachodontids in our consensus tree, the placement of Sinognathinae is extremely poorly supported, and taxon sampling in our analysis does not permit testing of the probainognathian hypothesis. Further research is required into the phylogenetic positions of the sinognathines, and indeed all the relationships within Gomphodontia. It is possible that convergent trait evolution is obscuring the correct placement of sinognathines. Until the recent description of *Etjoia*, sinognathines were the only taxa within Cynognathia, other than *Diademodon*, to possess conical anterior-most postcanines and they have an unusually slender snout in comparison to the rest of Cynognathia.

## Conclusions

*Guttigomphus avilionis* is the sixth known trirachodontid species, and along with the recently described *Impidens hancoxi* it presents a rapidly expanding understanding of the *Cricodon-Ufudocyclops* subzone of the Karoo Basin’s Burgersdorp Formation. All currently undisputed trirachodontids are known from South Africa, Tanzania, and Zambia. A broader geographic range for the Trirachodontidae is possible, but confirmation requires additional study of various poorly known extra-African taxa, most notably the Chinese Sinognathinae. The temporal range of trirachodontids is also currently questionable; although an Anisian age is usually cited for the main CAZ fauna, the possibility that the upper *Cricodon*-*Ufudocyclops* Subzone is later (Ladinian or even Carnian) cannot be excluded at present. Radioisotopic data for these strata are needed to test whether they are coeval with “*Cynognathus* Zone-like” faunas globally.

## Supplemental Information

10.7717/peerj.14355/supp-1 Supplemental Information 1 List of characters used for phylogenetic analysis.

10.7717/peerj.14355/supp-2 Supplemental Information 2 Character and character trait gradings for phylogenetic analysis.

10.7717/peerj.14355/supp-3 Supplemental Information 3 Raw data for analysis in TNT.

## Abbreviations

BP: Evolutionary Studies Institute (formerly the Bernard Price Institute for Palaeontological Research), University of the Witwatersrand, South Africa
BSPG: Bayerische Staatssammlung für Paläontologie und Geologie, Munich, Germany
NMQR: National Museum, Bloemfontein, South Africa
UMZC: University Museum of Zoology, Cambridge, UK

## Additional Information and Declarations

### Field Study Permissions

The following information was supplied relating to field study approvals (*i.e*., approving body and any reference numbers): The University of the Witwatersrand, Johannesburg, South Africa.

### Data Availability

The following information was supplied regarding data availability: The raw data used for phylogenetic analysis in TNT and characters and gradings are available in the Supplemental Files.

### New Species Registration

The following information was supplied regarding the registration of a newly described species: *Guttigomphus avilionis* LSID: urn:lsid:zoobank.org:act:57E697ED-DEC1-4E2D-A2E9-A6EC2E9A0FCA Publication LSID: urn:lsid:zoobank.org:pub:61EC40CA-DA44-4E1A-8316-84BCE847BE20
