# Supplementary material for: Guttigomphus avilionis gen. et sp. nov., a trirachodontid cynodont from the upper Cynognathus Assemblage Zone, Burgersdorp Formation of South Africa
Source: PeerJ. 2022 Dec 16;10:e14355. doi: 10.7717/peerj.14355 (PMC9762250; doi:10.7717/peerj.14355)
Supplement: Supplemental Information 1 [file peerj-10-14355-s001.docx]

Appendix 1

The characters and character traits used in this TNT analysis consist of a combination of the original data used by Abdala et al. 2006 and Sidor & Hopson 2018, with additions made by Kammerer 2008 and Gao et al 2010, along with minor modifications and additions as outlined below.

Incisors and canines (no data for incisors or canines is available for BP/1/5538 due to the incompleteness of the specimen):

1)  Upper incisor number: five or more (0); four (1); three (2). Abdala 2006 character 0 and Sidor & Hopson 2017 character 34. Ordered.

2)  Lower incisor number: four or more (0); three (1); two (2). Sidor and Hopson 2017 character 35. Ordered.

3)  Incisor size: small (0); some or all enlarged (1). Following Abdala 2006 character 1 and Sidor and Hopson 2017 character 37.

4)  Incisor procumbency: Absent (0); present (1). Added by Kammerer to Abdala’s original matrix in 2008, repeated by Gao et al 2010, character 44.

5)  Incisor cutting margins: serrated (0); smoothly ridged (1), denticulated (2). Abdala 2006 character 34, Sidor and Hopson 2017 character 36.

6)  Diastema between upper incisors and canine: present (0); absent (1). Abdala 2006 character 2.

7)  Upper canine size: large (0); reduced in size (1); absent (2). Abdala 2006 character 3, Sidor & Hopson 2017 character 38. Ordered.

8)  Lower canine size: large (0); reduced in size (1); absent (2). Abdala 2006 character 4, Sidor & Hopson character 39. Ordered.

9)  Canine serrations: Present (0); absent (1). Abdala 2006 character 35, Sidor & Hopson character 40. N.B: This character is graded the opposite way around by Sidor and Hopson with (0) marking absence and (1) marking presence.

10)  Position of the paracanine fossae in relation to the upper canine: anteromedial (0); medial (1); posteromedial (2). Abdala 2006 character 5, Sidor & Hopson character 2. Ordered.

Upper postcanines:

11) Diastema between upper canine and post-canines: (0) absent, (1) present, (2) enlarged. From Gao 3. Ordered.

12) First upper premolars: conical (0), sectorial (1), less transversely expanded gomphodont postcanines (2), none, all postcanines similar (3). Own addition.

13)  Size of the main cusps in the upper postcanines: labial lower than lingual (0); labial higher than lingual (1); even (2). Own addition following Abdala 2006 character 19. Comparison of the left lingual cusp and right buccal cusp of the most intact postcanines on BP/1/5538 suggests that the overall morphology has cusps of a similar height.

14)  Wider upper cusp in transeverse row: Symmetrical (0); lingual (1); buccal (2). Own addition following Sidor & Hopson character 56. BP/1/5538 is distinctly wider on the buccal cusp which is unique to this taxon.

15)  Overall morphology of the upper postcanines in occlusal view: sectorial (0); ovoid- elliptical (1); rectangular-trapezoidal (2). Abdala 2006 character 6.

16)  Transverse width of upper postcanines: Sectorial or around equal - up to 50% wider than long (0); Between 50-75% wider than long (1); between 75-100% wider than long (2); more than twice as wide as long with an excess of 100% difference (3). Own addition. Ordered.

17)  Number of cuspules around anterior and posterior border of crown: None (0); very faint cuspules only (1); Multiple pronounced cuspules (2). Own addition.

18)  Postcanine tooth row in adults: formed by sectorial (0); conical, gomphodont, and sectorial (1); gomphodont and sectorial (2). Abdala 2006 character 37, Gao et al amended.

19)  Shouldering in upper postcanines: absent (0); present (1). Abdala 2006 character 7.

20)  Inclination of the last upper postcanines in relation to the axis of the skull: absent or small (0); oblique (1). Abdala 2006 character 8.

21)  Position of the upper transverse cusp row on crown: on anterior half of crown (0); from midcrown almost to posterior margin (1); at posterior margin (no posterior cingulum). Sidor & Hopson 2017 character 44, Abdala 2006 character 9. Ordered.

22)  Number of upper cusps in transverse row: One (0); two (1); three or more (2). Abdala 2006 character 10, Sidor & Hopson 2017 character 43.

23)  Central cusp of upper transverse crest: absent (0); midway between buccal and lingual (1); closer to lingual cusp (2); closer to labial cusp (3). Abdala 2006 character 11 and Sidor & Hopson character 45 combined.

24)  Anterior cingulum in the upper postcanines: present (0); absent (1). Abdala 2006 character 39.

25)  Posterior cingulum on upper postcanines: present (0); absent (1). Abdala 2006. character 12.

26)  Upper postcanine buccal cingulum: absent (0); present (1). Sidor & Hopson 2017. character 41.

27)  Postcanine lingual cingulum: narrow (0); absent (1); lingually expanded (2). Sidor & Hopson 2017 character 42.

28)  Deep occlusal basins in the postcanines: absent (0); present (1). Abdala 2006 character 40.

29)  External cingulum on the anterior portion of the upper postcanines: absent (0); present (1). Abdala 2006 character 13.

30)  Anterolingual cusp in upper postcanines: absent (0); present (1). Abdala 2006 character 14, Sidor & Hopson 2017 character 79.

31)  Upper anterolabial accessory cusp: present (0); absent (1). Sidor & Hopson 2017 character 47.

32)  Upper posterolabial accessory cusp: present (0); absent (1). Sidor & Hopson 2017 character 48.

33)  Number of cusps in the sectorial border (i.e., labial margin) of the upper postcanines: (0) more than two; (1) two; (2) one. Abdala 2006 character 15.

34)  Upper anterior transverse (cingulum) ridge: low (0), high (1). Sidor and Hopson 2017 character 50.

35)  Longitudinal sheer surface of the main upper buccal cusp: anterior and posterior (to transverse ridge if present) (0); posterior only (1); anterior only (2). Sidor and Hopson 46.

36)  Number of posterior sectorial postcanines: six or more (0); three or four (1); one or two (2); none (gomphodont) (3). Sidor and Hopson 59. Ordered.

37)  Upper lingual ridge: absent (0); present (1). Sidor and Hopson 51. Lower postcanies:

38)  Overall morphology of lower postcanines in occlusal view: Sectorial (0); Circular (1); ovoid-elliptical (2); quadrangle (3). Abdala 16.

39)  Position of the transverse crest in lower postcanines: central (0); anterior (1). Abdala 17.

40)  Number of lower cusps in transverse row: one (0); two (1); three or more (2). Abdala 18, Sidor and Hopson 53.

41)  Size of the anterior cusps in the lower postcanines: labial lower than lingual (0); labial higher than lingual (1). Abdala 19.

42)  Anterior cingulum in lower postcanines: cuspules disposed on the entire margin (0); one or more cuspules located anterolabially (1); absent (2). Abdala 20.

43)  Lower anterior cingulum or cusp: present (0); absent (1). Sidor and Hopson 54.

44)  Posterior cingulum in the lower postcanines: present (0); absent (1). Abdala 38

45)  Posterior occlusal basin on lower postcanines: absent (0): present (1). Sidor and Hopson 55.

46)  Wider lower cusp in transverse row: Lingual (0); buccal (1). Sidor and Hopson 56.

47)  Shearing planes between the outer surface of the main cusp of the lower and the inner surface of the main cusp of the upper postcanines: present (0); absent (1). Abdala 42.

48)  Postcanine replacement pattern in adults: ‘alternating’ (0); widely spaced waves (three or more teeth per wave) (1); single wave (2). Sidor and Hopson 60.

Other cranial elements:

49)  Internarial bar: present (0); absent (1). Abdala 21

50)  Posterior portion of the maxillary tooth row inset from lateral margin of maxilla (cheek developed): absent (0); moderately set in (1); well set in (2). Sidor and Hopson 57. Ordered.

51)  Axis of the posterior part of maxillary tooth row: directed towards the lateral rim of the subtemporal fenestra (0); directed towards the centre of the fenestra (1); directed toward the medial rim of the fenestra (2). Abdala 36. Repeated by Sidor and Hopson (58).

52)  Extent of maxillary tooth row: essentially antorbital (0); extending substantially suborbitally (1). Gao et, al 2010 character 45.

53)  Length of secondary palate relative to anterior border of the orbit: shorter (0); about equal (1); longer (2). Gao et, al character 46, who modified it from Hopson and Kitching 2001, repeated by Sidor and Hopson (character 10). Ordered.

54)  Posterior border of secondary palate in relation to maxillary tooth row: close to midlevel of postcanine row (0); close to posterior end of tooth row (1). Modified by Gao et, al. from Hopson and Kitching (2001) character 50, similar to Sidor and Hopson’s character 9.

55)  Major palatine foramen at maxillary/palatine suture (0); penetrating palatine (1). Kielan-Jaworowska et, al. 2004, repeated by Gao et, al. 2010 character 48 and similar to Sidor and Hopson’s Character 1.

56)  Extent of the contribution of the palatine to secondary bony osseous palate: far less than one-third of the palate (0); more than one-third of the palate (1). Kielan- Jaworowska et, al. 2004, repeated by Gao et, al. 2010 character 49.

57)  Parietal foramen in adults: present (0); absent (1). Abdala 2006 character 23.

58) ‘V’-shaped notch separating the lambodial crest from the zygomatic arch: absent (0); present (1). Sidor and Hopson character 26.

59)  Zygomatic process of the jugal: slightly projected (0); conspicuously projected (1); absent (2). Abdala 2006 character 24.

60)  Posterior extension of the jugal above the squamosal in the zygoma: absent or with a small extension (0); well developed (1). Abdala 2006 character 25.

61)  Zygomatic arch, dorsoventral height: slender (0); moderately deep (1); very deep (2). Sidor and Hopson character 12.

62)  Greatest width of zygomatic arch: about middle of arch (anterior to level of quadrate) (0); at posterior end of arch (at level of quadrate) (1). Sidor and Hopson 2017 character 23.

63)  Jugal depth in zygomatic arch relative to exposed squamosal depth: less than twice (0); greater than twice (1). Sidor and Hopson 2017 character 14.

64)  Jugal suborbital process: absent (0); present (1). Sidor and Hopson 2017 character 15.

65)  Coronoid process of the mandible: covers last postcanine in lateral view (0); does not cover last postcanine (1). Abdala 2006 character 26.

66)  Dentary angle: Not projecting posteriorly (0); weakly projecting (1); well projecting posteriorly (2). Abdala 2006 character 27. Ordered.

67)  Snout: Longer than temporal region (0); subequal (1); Substantially shorter than temporal region (2). Abdala 2006 character 28. Ordered.

68)  Premaxilla forms posterior border of incisive foramen: absent (0); present (1). Sidor and Hopson character 1 (but see also character 54 of this matrix)

69)  Ectopterygoid: present (0); absent (1). Abdala 2006 character 30.

70)  Maxilla in margin of the subtemporal fenestra: excluded (0); included (1). Abdala 2006 character 31.

71)  Epipterygoid-quadrate contact: present (0); squamosal interposed (1). Abdala 2006 character 32.

72)  Temporal fenestra: widest posteriorly (0); same width throughout (1); widest in the middle (2). Abdala 2006 character 33.

73)  Intertemporal crest in dorsal view: extending to or close to posterior border of temporal fenestra (0); shortening anteriorly with obvious posterior bifurcation (1). Gao et, al 2010 character 47

74)  Ventral surface of basisphenoid depressed below occipital condyles: less than 1⁄4 occipital height (0); greater than 1⁄4 occipital height (1). Sidor and Hopson 2017 character 11.

75)  Squamosal groove for external auditory meatus: shallow (0); moderately deep (1); very deep (2). Sidor and Hopson 2017 character 16.

76)  Frontal-palatine contact in orbit: absent (0); present (1). Sidor and Hopson 2017 character 17.

77)  Descending flange of the squamosal lateral to the quadratojugal: absent (0); present, not contacting surangular (1); present, contacting surrangular (2). Sidor and Hopson 2017 character 18.

78)  Internal carotid foramina in basisphenoid: present (0); absent (1). Sidor and Hopson 2017 character 19.

79)  Groove on prootic extending from pterygoparoccipital foramen to trigeminal foramen: present and open (0); present and closed as a canal (1). Sidor and Hopson 2017 character 20.

80)  Trigeminal nerve exit: between prootic incisura and epipterygoid (0); via foramen between prootic and epipterygoid (1); via two foramina (2). Sidor and Hopson 2017 character 21.

81)  Postdentary rod height relative to exposed length (distance between base of reflected lamina and jaw joint): greater than 1⁄2 length (0); about 1⁄2 length (1); less than 1⁄2 length (2). Sidor and Hopson 2017 character 31. Ordered.
