# Supplementary material for: Guttigomphus avilionis gen. et sp. nov., a trirachodontid cynodont from the upper Cynognathus Assemblage Zone, Burgersdorp Formation of South Africa
Source: PeerJ. 2022 Dec 16;10:e14355. doi: 10.7717/peerj.14355 (PMC9762250; doi:10.7717/peerj.14355)
Supplement: Supplemental Information 2 [file peerj-10-14355-s002.docx]

Appendix 2

| **Taxa** | **05** | **10** | **15** | **20** | **25** | **30** | **35** | **40** | **45** | **50** |
| --- | --- | --- | --- | --- | --- | --- | --- | --- | --- | --- |
| *Cynognathus* | 11000 | 00000 | 00??0 | 000?0 | ?00?? | 0100? | ??0?0 | 0?0?0 | ??0?0 | 0??00 |
| *Diademodon* | 11000 | 00000 | 10??1 | 0?100 | 12100 | 02000 | 00000 | 10101 | ?0000 | 00100 |
| *Trirachodon Kanne.* | 11000 | 00000 | 12201 | 32200 | 12100 | 02??0 | 00200 | 21202 | ?0000 | ?1102 |
| *Trirachodon berryi* | 1100? | 00000 | 0220? | 323?? | 121?? | 02000 | 00?00 | 31??2 | ??0?0 | ??1?2 |
| *Langbergia modisei* | 11001 | 00000 | 22??1 | 0?200 | 12100 | 02000 | 00000 | 11202 | ?0000 | ?1100 |
| *Cricodon* | 11100 | 00000 | 01111 | 22200 | 12100 | ??10? | 00200 | 21202 | ?0000 | ?1102 |
| *Andescynodon* | 11001 | 00010 | 02112 | 20300 | 01?11 | 12100 | ??102 | ??311 | 11?11 | ?0?0? |
| *Massetognathus* | 11002 | 11111 | 12202 | ??31? | 22201 | 02101 | 10002 | 31311 | 02101 | 00202 |
| *Exaeretodon* | 21111 | 10112 | 2??02 | 3?311 | 21011 | 02101 | 11012 | 31311 | 12111 | 10212 |
| *Luangwa* | 11000 | 00000 | 10??1 | ??300 | 12200 | 12110 | 10?02 | 31311 | 01001 | 00202 |
| *Scalenodon ang.* | 11000 | 00000 | ??201 | 11300 | 12211 | 12110 | 00202 | 30311 | 01001 | 00201 |
| *Mandagomphodon* | 22101 | 11?10 | 13?02 | 1030? | 12200 | 02101 | 10102 | 31311 | 01001 | 00202 |
| *Traversodon* | 1?001 | 00010 | 0???2 | ??311 | 22200 | ??111 | ??1?? | ??311 | 02??? | ?0?0? |
| *Gomphodontosuchus* | 11001 | 11111 | ????2 | 0?311 | 21011 | 02101 | 10?12 | 31311 | 12111 | 10202 |
| *Pascualgnathus* | 21001 | 00010 | 00101 | 10300 | 01000 | 021?0 | 01201 | 11311 | ????1 | 002?1 |
| *Scalenodontoides* | 21101 | 1???2 | 13102 | 1031? | 21?11 | ??10? | ??00? | 31311 | 12?1? | ?0?12 |
| *Menadon* | 1?111 | 11101 | 20??2 | 00311 | 21?11 | ??101 | ??2?? | ??311 | ?2?1? | ?0?01 |
| *Dadadon* | 11001 | 01110 | 22102 | 30311 | 22211 | ??111 | ??20? | 3?3?1 | 11??? | ?0?01 |
| *Santacruzodon* | 1?002 | 0??10 | ?2102 | 20311 | 22201 | ??100 | ??0?? | 3?311 | 12?01 | ?1??1 |
| *Sinognathus* | 120?1 | 00010 | 10??1 | ?0101 | 121?0 | 02?00 | 00?00 | 312?1 | ??02? | ?1??1 |
| *Beishanodon* | 1???1 | 000?0 | 00?01 | 1?101 | 223?0 | 02000 | 0000? | 31??? | ????? | ???01 |
| *Guttigomphus* | ????? | ????? | ??221 | 212?0 | 12101 | 121?0 | 00000 | 21??? | ????? | ????1 |
| *Etjoia dentitransitus* | 21012 | 00002 | 00011 | 2210? | 1220? | ??1?0 | 00010 | 11211 | 0?0?1 | ?0??0 |
| *Impidens hancoxi* | ??1?? | 00??0 | 12?11 | 1?211 | ????? | ????? | ????? | 0???? | ????? | ????1 |
| **Taxa** | **55** | **60** | **65** | **70** | **75** | **80** | **81** |  |  |  |
| *Cynognathus* | 00000 | 00000 | 21010 | 00000 | 00002 | 02101 | 2 |  |  |  |
| *Diademodon* | 00010 | 00110 | 2111? | 00000 | 00102 | 02102 | 2 |  |  |  |
| *Trirachodon Kanne.* | 20100 | 00110 | 21110 | 01?00 | 01102 | 02112 | 2 |  |  |  |
| *Trirachodon berryi* | 2?10? | ?01?? | 21111 | ????? | ???0? | 0???? | ? |  |  |  |
| *Langbergia modisei* | 11100 | 00110 | 21111 | 01000 | 01002 | ?2??2 | 1 |  |  |  |
| *Cricodon* | 10?0? | ?01?0 | ????1 | 0???0 | ????2 | ?2??? | 2 |  |  |  |
| *Andescynodon* | 2???? | ?1120 | ????? | 010?? | ?0??? | ????? | ? |  |  |  |
| *Massetognathus* | 21201 | 01120 | 21100 | 01110 | 11112 | 02112 | 2 |  |  |  |
| *Exaeretodon* | 21100 | 01111 | 21010 | 20?11 | 111?2 | ?2112 | 2 |  |  |  |
| *Luangwa* | 20??? | ?0110 | 21110 | 10??0 | ?1??2 | 02112 | 2 |  |  |  |
| *Scalenodon ang.* | 2?0?? | ?0121 | 21110 | ?10?0 | ?0?12 | 02112 | 2 |  |  |  |
| *Mandagomphodon* | 2?1?? | ??1?? | ??1?? | ??01? | ????2 | ?2??? | 2 |  |  |  |
| *Traversodon* | 2???? | ?011? | ????0 | ????0 | ????? | ????? | ? |  |  |  |
| *Gomphodontosuchus* | 2?0?? | ????? | ????0 | ????0 | ????2 | 021?? | 2 |  |  |  |
| *Pascualgnathus* | 211?? | ?1100 | 21110 | 01??0 | ?0?02 | 02112 | 2 |  |  |  |
| *Scalenodontoides* | 2???? | ?11?1 | ????0 | 21??1 | ?2??? | ????? | ? |  |  |  |
| *Menadon* | 21??? | ??121 | ???10 | 20??0 | ????? | ????? | ? |  |  |  |
| *Dadadon* | 21??? | ??13? | ?0??? | ????? | ????? | ????? | ? |  |  |  |
| *Santacruzodon* | 21??? | ??13? | ???00 | 0???? | ????? | ????? | ? |  |  |  |
| *Sinognathus* | 1011? | ?111? | 20?00 | 00??? | ??002 | ????? | 2 |  |  |  |
| *Beishanodon* | 20111 | 1111? | 10000 | ??000 | 00101 | ????? | ? |  |  |  |
| *Guttigomphus* | ?0??? | ????? | ???1? | ????? | ????? | ????? | ? |  |  |  |
| *Etjoia dentitransitus* | ?1??? | ?011? | 2011? | 01??0 | ?1??2 | ????? | ? |  |  |  |
| *Impidens hancoxi* | 1010? | ????? | ????? | ????0 | ?0??? | ????? | ? |  |  |  |
